# RAB31 marks and controls an ESCRT-independent exosome pathway

**DOI:** 10.1038/s41422-020-00409-1

**Published:** 2020-09-21

**Authors:** Denghui Wei, Weixiang Zhan, Ying Gao, Liyan Huang, Run Gong, Wen Wang, Ruhua Zhang, Yuanzhong Wu, Song Gao, Tiebang Kang

**Affiliations:** 1 State Key Laboratory of Oncology in South China, Collaborative Innovation Center for Cancer Medicine, Sun Yat-sen University Cancer Center, Guangzhou, Guangdong 510060 China; 2 Department of Abdominal Oncology, The Cancer Center of the Fifth Affiliated Hospital of Sun Yat-sen University, Zhuhai, Guangdong 519000 China

**Keywords:** Small GTPases, Endosomes, Multivesicular bodies, Lysosomes, ESCRT

## Abstract

Exosomes are generated within the multivesicular endosomes (MVEs) as intraluminal vesicles (ILVs) and secreted during the fusion of MVEs with the cell membrane. The mechanisms of exosome biogenesis remain poorly explored. Here we identify that RAB31 marks and controls an ESCRT-independent exosome pathway. Active RAB31, phosphorylated by epidermal growth factor receptor (EGFR), engages flotillin proteins in lipid raft microdomains to drive EGFR entry into MVEs to form ILVs, which is independent of the ESCRT (endosomal sorting complex required for transport) machinery. Active RAB31 interacts with the SPFH domain and drives ILV formation via the Flotillin domain of flotillin proteins. Meanwhile, RAB31 recruits GTPase-activating protein TBC1D2B to inactivate RAB7, thereby preventing the fusion of MVEs with lysosomes and enabling the secretion of ILVs as exosomes. These findings establish that RAB31 has dual functions in the biogenesis of exosomes: driving ILVs formation and suppressing MVEs degradation, providing an exquisite framework to better understand exosome biogenesis.

## Introduction

Extracellular vesicles (EVs) are a heterogeneous group of cell-derived membranous structures mainly comprising exosomes and microvesicles, which originate from the endosomal system and are shed from the plasma membrane, respectively.^1^ Exosomes are present in biological fluids and function in intercellular communication, allowing cells to exchange proteins, lipids, genetic materials, amino acids and metabolites.^2–7^ Exosomes are generated as intraluminal vesicles (ILVs) within the lumen of endosomes during their maturation into multivesicular endosomes (MVEs) and secreted by the fusion of MVEs with the cell surface.^1,8^ The formation of ILVs by the inward budding of MVEs is mostly mediated by the ESCRT (endosomal sorting complex required for transport) machinery,^1,8,9^ as many cargoes, including currently well-known syndecan, tetraspanin CD63, and Toll-like receptor trafficking chaperone UNC93B1 etc., recruit Syntenin-Alix-ESCRT-III pathway by the cytoplasmic tails to mediate their ILV formation.^1,10–13^ Although ESCRTIII is always considered to be required for the scission of the ILVs into the MVE lumen,^1^ ILVs within the lumen of MVEs are still formed in the ESCRT-depleted cells, indicating that the ESCRT-independent pathways for ILV formation exist.^9^ Indeed, the first ESCRT-independent mechanism for ILV biogenesis was shown to require sphingolipid ceramide, which may allow the generation of raft-based microdomains inducing a spontaneous negative curvature on the membranes.^14^ However, which proteins are needed and how they function in this ESCRT-independent ILV formation remain unknown.

Prior to the fusion of MVEs with the cell surface towards exosome secretion, a key checkpoint must suppress ILVs degradation by preventing the fusion of MVEs with lysosomes.^1^ The accumulated nondegradable MVEs use the common secretory machineries for exosome secretion, which is mainly regulated by RAB27.^1,15^ Therefore, exosome biogenesis pathway mainly contains three key steps accompanied by endosomal vesicular transport: ILV formation, prevention of MVEs degradation and the fusion of MVEs with the cell surface.^1^ The regulatory mechanism of the balance between degradative and secretory capacity of MVEs remains largely unexplored.^1^ Many membrane proteins have been detected in exosomes that are involved in immune responses, viral infection, metabolic and cardiovascular diseases, neurodegenerative diseases and cancer progression,^1,7,16^ but the regulatory machineries for their sorting into exosomes are still mysterious. Endocytic membrane proteins, particularly the receptor tyrosine kinase (RTK) family including epidermal growth factor receptor (EGFR),^17,18^ are targeted to endosomes and MVEs, and are destined to lysosomes for degradation by the fusion of MVEs with lysosomes,^19–22^ which are mediated by multiple RAB GTPases and the ESCRT machinery.^19,21,23–26^ The ESCRT machinery sorts the ubiquitylated EGFR into ILVs for lysosomal degradation,^19,20,23,27^ which is defined as the canonical model for endolysosomal sorting of membrane proteins in the MVE pathway.^21,22^ In fact, EGFR is frequently accumulated and/or mutated in multiple types of cancer,^17,18,28,29^ and is present in exosomes derived from cancer cell lines and patient serum.^30–34^ This phenomenon suggests that the sorting machinery for EGFR into ILVs towards exosome secretion may be different from the ESCRT machinery in two key steps, ILV formation and prevention of MVEs degradation. This process may be also regulated by RAB GTPase members, as RAB GTPases are localized on the surface of specific membranes and regulate their vesicular transport through the recruitment of specific effector proteins.^19,24,26^ For instance, in the endolysosomal transport network, RAB5 regulates the formation and fusion of early endosomes;^24,35,36^ The RAB5-RAB7 conversion regulates the transition from early to late endosomes;^24,37,38^ RAB7 regulates the fusion of late endosomes/MVEs with lysosomes to degrade the ILVs;^19,27^ RAB27 regulates the docking and fusion of MVEs with plasma membrane to secrete the ILVs as exosomes.^1,15^

In this study, we found that active RAB31 drives EGFR entry into MVEs to form ILVs and exosomes, and that EGFR, perhaps other RTKs, phosphorylates RAB31 to drive the formation of cognate exosomes. Flotillin proteins in lipid raft microdomains are engaged in this ILV formation driven by active RAB31, which is independent of the ESCRT machinery. We further demonstrated that RAB31 recruits TBC1D2B to inactivate RAB7 to suppress the fusion of MVEs with lysosomes and to enable the secretion of exosomes. These findings establish an ESCRT-independent exosome pathway that is marked and controlled by RAB31, shedding light on the better understanding of the heterogeneous biogenesis of exosomes.

## Results

### Active RAB31 directs EGFR localization to CD63-positive MVEs

**Fig. 1:**
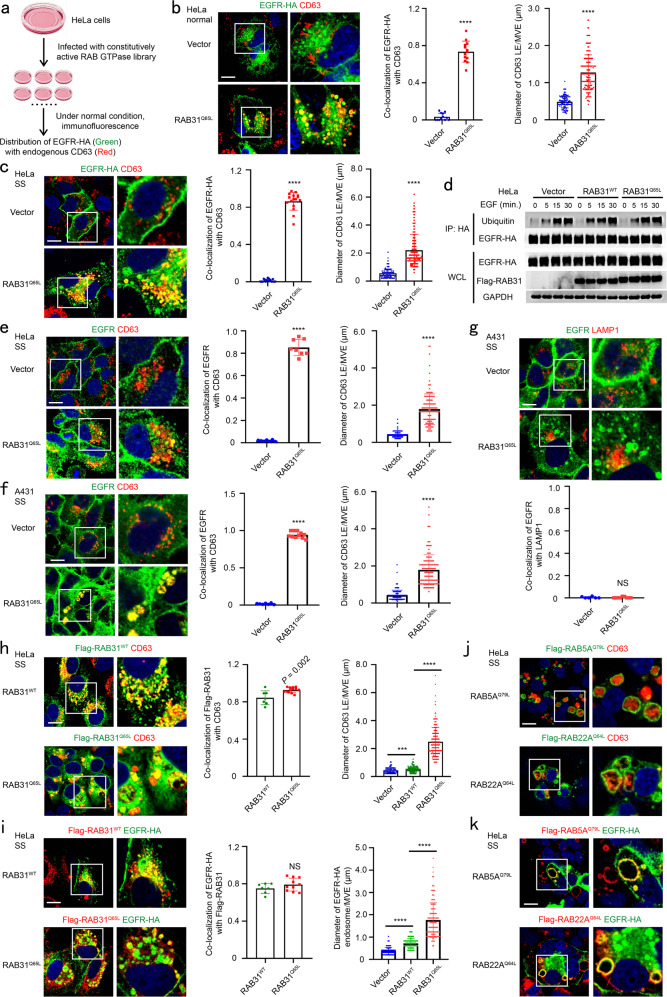
Active RAB31 directs EGFR localization to enlarged CD63-positive MVEs. **a** Schematic depicting the workflow for the screening method. **b** Left, immunofluorescence of EGFR-HA (green) and CD63 (red) in the indicated stable HeLa cells transiently expressing EGFR-HA under normal condition. Middle, the ratio of co-localization of EGFR-HA with CD63-positive late endosome and MVE (LE/MVE) in Vector (*n* = 12 fields) and RAB31^Q65L^ (*n* = 13 fields). Right, diameter of CD63-positive LE/MVE in Vector (*n* = 120) and RAB31^Q65L^ (*n* = 150). **c** Left, immunofluorescence of EGFR-HA (green) and CD63 (red) in the indicated stable HeLa cells transiently expressing EGFR-HA under serum starvation (SS). Middle, the ratio of co-localization of EGFR-HA with CD63-positive LE/MVE in Vector (*n* = 16 fields) and RAB31^Q65L^ (*n* = 16 fields). Right, diameter of CD63-positive LE/MVE in Vector (*n* = 150) and RAB31^Q65L^ (*n* = 180). **d** Western blotting analyses of whole-cell lysates (WCL) and immunoprecipitates (IP) from the indicated stable HeLa cells under SS upon EGF treatment for the indicated time points. **e** Left, immunofluorescence of endogenous EGFR (green) and CD63 (red) in the indicated stable HeLa cells under SS. Middle, the ratio of co-localization of EGFR with CD63-positive LE/MVE in Vector (*n* = 7 fields) and RAB31^Q65L^ (*n* = 8 fields). Right, diameter of CD63-positive LE/MVE in Vector (*n* = 142) and RAB31^Q65L^ (*n* = 147). **f** Left, immunofluorescence of endogenous EGFR (green) and CD63 (red) in the indicated stable A431 cells under SS. Middle, the ratio of co-localization of EGFR with CD63-positive LE/MVE in Vector (*n* = 7 fields) and RAB31^Q65L^ (*n* = 11 fields). Right, diameter of CD63-positive LE/MVE in Vector (*n* = 150) and RAB31^Q65L^ (*n* = 154). **g** Up panels, immunofluorescence of endogenous EGFR (green) and LAMP1 (red) in the indicated stable A431 cells under SS. Low panel, the ratio of co-localization of EGFR with LAMP1-positive lysosome in Vector (*n* = 6 fields) and RAB31^Q65L^ (*n* = 9 fields). **h** Left, immunofluorescence of Flag-RAB31 (green) with CD63 (red) in the indicated stable HeLa cells under SS. Middle, the ratio of co-localization of Flag-RAB31 with CD63-positive LE/MVE in RAB31^WT^ (*n* = 7 fields) and RAB31^Q65L^ (*n* = 13 fields). Right, diameter of CD63-positive LE/MVE in Vector (*n* = 150), RAB31^WT^ (*n* = 165) and RAB31^Q65L^ (*n* = 180). **i** Left, immunofluorescence of Flag-RAB31 (red) with EGFR-HA (green) in the indicated stable HeLa cells transiently expressing EGFR-HA under SS. Middle, the ratio of co-localization of Flag-RAB31 with EGFR-HA-positive vesicle in RAB31^WT^ (*n* = 7 fields) and RAB31^Q65L^ (*n* = 10 fields). Right, diameter of EGFR-HA-positive vesicle in Vector (*n* = 129), RAB31^WT^ (*n* = 139) and RAB31^Q65L^ (*n* = 179). **j** Immunofluorescence of Flag-RAB5A^Q79L^ and Flag-RAB22A^Q64L^ (green) with CD63 (red) in the indicated stable HeLa cells under SS. **k** Immunofluorescence of Flag-RAB5A^Q79L^ and Flag-RAB22A^Q64L^ (red) with EGFR-HA (green) in the indicated stable HeLa cells transiently expressing EGFR-HA under SS. All data are means ± SD. Unpaired *t*-test was used to analyze the difference between the two groups. ****P < 0.0001, NS, no statistical significance. Scale bars, 10 μm.

To identify the RAB GTPases regulating EGFR sorted into ILVs for exosome secretion, a library including 62 constitutively active forms of RAB GTPases tagged with Flag was generated, and their individual stable HeLa cell lines were also generated (Supplementary information, Fig. S1a). Then, we detected the co-localization of EGFR with endogenous CD63, the well-known marker of late endosomes and MVEs (LE/MVEs),^15^ and found that only the constitutively active form of RAB31 (RAB31^Q65L^, Glutamine mutated to Leucine), but not those of other RAB members, specifically directed hemagglutinin (HA)-tagged EGFR (EGFR-HA) localization to the enlarged CD63-positive MVEs (Fig. 1a, b; Supplementary information, Fig. S1b). Interestingly, this localization of EGFR to the enlarged CD63-positive MVEs driven by RAB31^Q65L^ was also observed under serum starvation (Fig. 1c). As expected, ubiquitylation of EGFR-HA was not detected under serum starvation (0 point of Fig. 1d), indicating that ubiquitylation for EGFR transport and localization is not involved in this phenomenon induced by RAB31^Q65L.^^23^ In addition, RAB31 had no effect on the ubiquitylation of EGFR upon EGF treatment (5, 15, 30 min points of Fig. 1d). Moreover, EGFR-HA was also co-localizated with EEA1, an early endosome marker, but neither LAMP1, a lysosomal marker, nor GFP-tagged LC3 puncta, an indicator of the autophagosomes induced by serum starvation (Supplementary information, Fig. S2a–c), suggesting that serum starvation does not affect the endocytosis of EGFR and this endocytic EGFR is localized to CD63-positive MVEs driven by RAB31^Q65L^ rather than transported to lysosomes. More importantly, RAB31^Q65L^ directed endogenous EGFR localization to the enlarged CD63-positive MVEs, but not LAMP1-positive lysosomes (Fig. 1e–g). Even though both wild-type (WT) RAB31 (RAB31^WT^) and RAB31^Q65L^ co-localized with CD63 and EGFR, only RAB31^Q65L^ entered the enlarged CD63-positive MVEs (Fig. 1h, i). Although RAB5A, RAB22A and RAB31 belong to the RAB5 subfamily,^39^ both RAB5A^Q79L^ and RAB22A^Q64L^ were only localized on the membranes of enlarged endosomes, where EGFR was also localized (Fig. 1j, k), indicating that RAB31^Q65L^ is unique in triggering the budding of MVE membranes in the RAB GTPase family. This phenomenon was observed in multiple human cancer cell lines (Supplementary information, Fig. S2d). Many mutants of RAB31 have been detected in various types of human cancer (Supplementary information, Fig. S2e), and we were very curious to test whether any RAB31 mutant may also induce this localization of EGFR to CD63-positive MVEs. Fascinatingly, G13W, R25Q, Q28H, P37H, A41P, S42F, G64V, R67L, R77Q, K135N, A138T, F160C and R165S (13 of 45 mutations), but not other mutants, could also direct EGFR localization to the enlarged CD63-positive MVEs, whereas RAB31^WT^ only directed EGFR to the normal-sized CD63-positive late endosomes (Supplementary information, Fig. S2f), indicating that these 13 RAB31 mutants can function similarly to the active form RAB31^Q65L^. Taken together, these results demonstrate that active RAB31 directs EGFR to the enlarged CD63-positive MVEs.

### Active RAB31 drives EGFR entry into CD63-positive MVEs to form ILVs and exosomes

**Fig. 2:**
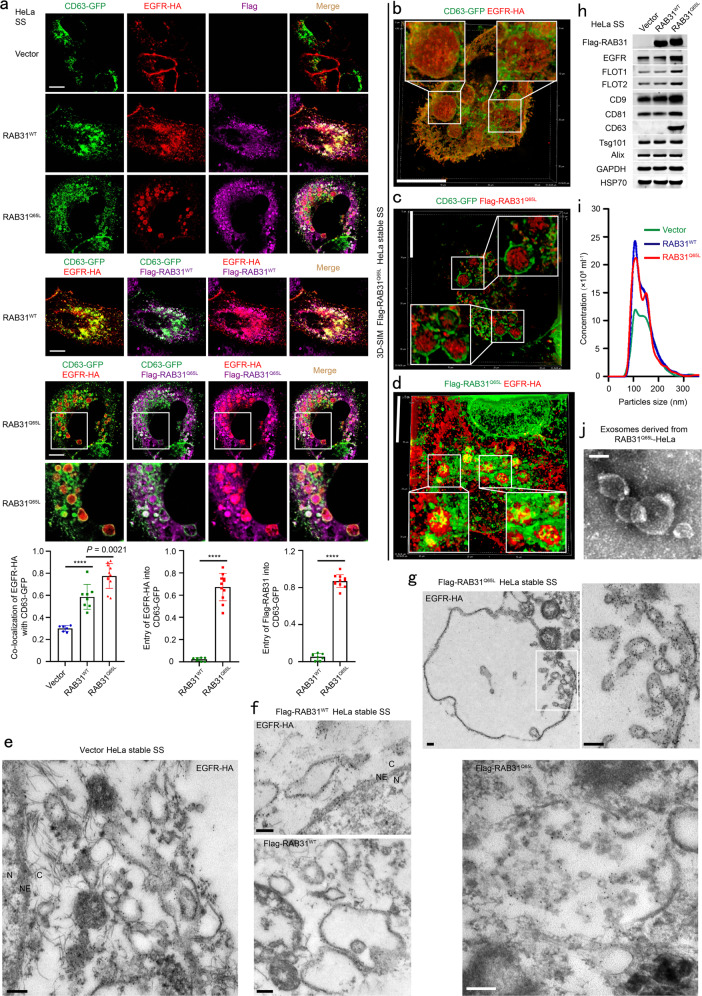
Active RAB31 drives EGFR entry into CD63-positive MVEs to form ILVs and exosomes. **a** Up panels, immunofluorescence of EGFR-HA (red) and Flag-RAB31 (magenta) with CD63-GFP (green) in the indicated stable HeLa cells transiently expressing EGFR-HA and CD63-GFP under serum starvation (SS). Low panel left, the ratio of co-localization of EGFR-HA with CD63-GFP-positive LE/MVE in Vector (*n* = 6 fields), RAB31^WT^ (*n* = 8 fields) and RAB31^Q65L^ (*n* = 11 fields). Low panel middle, the ratio of entry of EGFR-HA into CD63-GFP-positive LE/MVE in RAB31^WT^ (*n* = 8 fields) and RAB31^Q65L^ (*n* = 11 fields). Low panel right, the ratio of entry of Flag-RAB31 into CD63-GFP-positive LE/MVE in RAB31^WT^ (*n* = 8 fields) and RAB31^Q65L^ (*n* = 11 fields). **b**–**d** Immunofluorescence of the localization of EGFR-HA (red) with CD63-GFP (green) (**b**), Flag-RAB31^Q65L^ (red) with CD63-GFP (green) (**c**), and Flag-RAB31^Q65L^ (green) with EGFR-HA (red) (**d**) in Flag-RAB31^Q65L^ stable HeLa cells transiently expressing EGFR-HA and CD63-GFP under SS using three-dimensional structured illumination microscopy (3D-SIM). **e** Immunoelectron microscopy of the localization of EGFR-HA in Vector stable HeLa cells transiently expressing EGFR-HA under SS. NE, nuclear envelope; N, nucleus; C, cytoplasm. **f** Immunoelectron microscopy of the localization of EGFR-HA and Flag-RAB31^WT^ in Flag-RAB31^WT^ stable HeLa cells transiently expressing EGFR-HA under SS. NE, nuclear envelope; N, nucleus; C, cytoplasm. **g** Immunoelectron microscopy of the localization of EGFR-HA and Flag-RAB31^Q65L^ in Flag-RAB31^Q65L^ stable HeLa cells transiently expressing EGFR-HA under SS. **h** Western blotting analyses of the concentrated conditional media from the indicated stable HeLa cells under SS. **i** NanoSight nanoparticle tracking analysis of the concentrated conditional media from the indicated stable HeLa cells under SS. **j** Transmission electron microscopy analysis of the concentrated conditional media from Flag-RAB31^Q65L^ stable HeLa cells under SS. All data are means ± SD. Unpaired *t*-test was used to analyze the difference between the two groups. ****P < 0.0001. Scale bars, 10 μm (**a**–**d**), 200 nm (**e**–**g**), 100 nm (**j**).

Using Flag-RAB31, EGFR-HA and GFP-tagged CD63 (CD63-GFP), we simultaneously investigated their distributions. Both RAB31^Q65L^ and EGFR-HA entered the enlarged CD63-positive MVEs (Fig. 2a), and they were simultaneously localized on ILVs, as shown by super-resolution structured illumination microscopy (SIM) (Supplementary information, Fig. S2g). Strikingly, numerous ILVs bearing EGFR-HA, Flag-RAB31^Q65L^, or both were clearly observed in CD63-positive MVEs using three-dimensional SIM (3D-SIM) (Fig. 2b–d). Next, immunoelectron microscope (IEM) was used to further investigate the precise localization of EGFR and RAB31 on LE/MVEs. The IEM results clearly showed that EGFR-HA was localized to the membrane of endosomes in Vector and Flag-RAB31^WT^ cells (Fig. 2e, f), whereas EGFR-HA was localized to the membrane of MVEs and ILVs in Flag-RAB31^Q65L^ cells (Fig. 2g). As expected, Flag-RAB31^WT^ was localized to the surface of late endosomes (Fig. 2f), whereas Flag-RAB31^Q65L^ was localized to the membrane of ILVs (Fig. 2g). These results determine that RAB31^Q65L^ but not RAB31^WT^ drives EGFR entry into MVEs to form ILVs under serum starvation. Interestingly, both RAB31^WT^ and RAB31^Q65L^ could increase the number of particles by NanoSight nanoparticle tracking analysis, but only RAB31^Q65L^ markedly increased EGFR protein in the concentrated conditional media mainly containing exosomes (Fig. 2h, i), and such exosomes were validated with a spherical appearance by transmission electron microscopy (Fig. 2j). Notably, some well-known EV markers, such as Flotillin1 (FLOT1), Flotillin2 (FLOT2), CD9, CD81, and CD63^1,40,41^ were also significantly increased by RAB31^Q65L^ in the concentrated conditional media but not in cells (Fig. 2h; Supplementary information, Fig. S3a), whereas the ESCRT-associated EV markers, such as Tsg101 and Alix,^1,40^ were not altered in the same concentrated conditional media (Fig. 2h), suggesting that the ESCRT machinery may not be involved in the formation of EGFR-containing exosomes driven by RAB31^Q65L^. Together, these results show that active RAB31 drives EGFR entry into CD63-positive MVEs to form ILVs and to promote the production of EGFR-containing exosomes.

Next, we further employed high-resolution density gradient fractionation^6^ to separate small extracellular vesicles (sEVs) and non-vesicular (NV) extracellular matter in the concentrated conditional media from both NCI-H1975 and MDA-MB231 cells that have higher levels of endogenous RAB31 compared to HeLa cells (Supplementary information, Fig. S3b). The high enrichment of endogenous RAB31, FLOT1, FLOT2 and EGFR in sEV fraction pools was validated using this method, as the classical EV markers CD9, CD81, CD63, Syntenin-1, Alix, Tsg101 and VPS4 were also highly enriched in the same pools (Supplementary information, Fig. S3c). Meanwhile, non-membrane proteins GAPDH, HSP70, HSP90, β-tubulin, β-actin and Histone H3 were highly enriched in NV fractions from MDA-MB231 cells, whereas these proteins were slightly enriched in EV fractions and also distributed in NV fractions from NCI-H1975 cells (Supplementary information, Fig. S3c). These results suggest that membrane proteins are preferentially secreted into exosomes and other EVs rather than into NV extracellular matter, although the distribution of these non-membrane proteins is distinct in these two cell lines.

### Active RAB31 engages FLOTs to drive EGFR-containing ILV formation depending on cholesterol and ceramide in lipid raft microdomains

Indeed, we further showed that ESCRT components Hrs and Tsg101, as well as Alix^1,10,23^ were not involved in the production of EGFR-containing exosomes driven by RAB31^Q65L^, baucase the EGFR protein level in the concentrated conditional media was not affected by knocking down of these molecules using two short hairpin RNAs (shRNAs) (Supplementary information, Fig. S4a, b), suggesting that the formation of exosomes driven by RAB31^Q65L^ is separated from that of exosomes driven by ESCRT. Moreover, knocking down tetraspanins CD9 or CD81 did not alter these functions of RAB31^Q65L^ (Supplementary information, Fig. S4c–e), although CD9 and CD81 were increased in the concentrated conditional media driven by RAB31^Q65L^ (Fig. 2h). Interestingly, knockdown of CD63 decreased the protein levels of CD9, CD81, EGFR and Flag-RAB31^Q65L^ in cells (Supplementary information, Fig. S4f), and also decreased these proteins as well as FLOT1 and FLOT2 in the concentrated conditional media (Supplementary information, Fig. S4g). However, knockdown of CD63 did not change the entry of Flag-RAB31^Q65L^ and EGFR-HA into Hrs-positive MVEs (Supplementary information, Fig. S4h). These results suggest that ESCRT, CD9 and CD81 are not required for the production of EGFR-containing exosomes driven by RAB31^Q65L^. We noted that depletion of CD63 may influence the structure and appearance of late endosomes and MVEs (Supplementary information, Fig. S4h), as CD63 is the main component of late endosomes and MVEs. This may explain why the decrease of some membrane proteins mentioned above from both cells and media were observed in cells with CD63 depletion. Therefore, we propose that active RAB31 marks an ESCRT-independent exosome pathway.

**Fig. 3:**
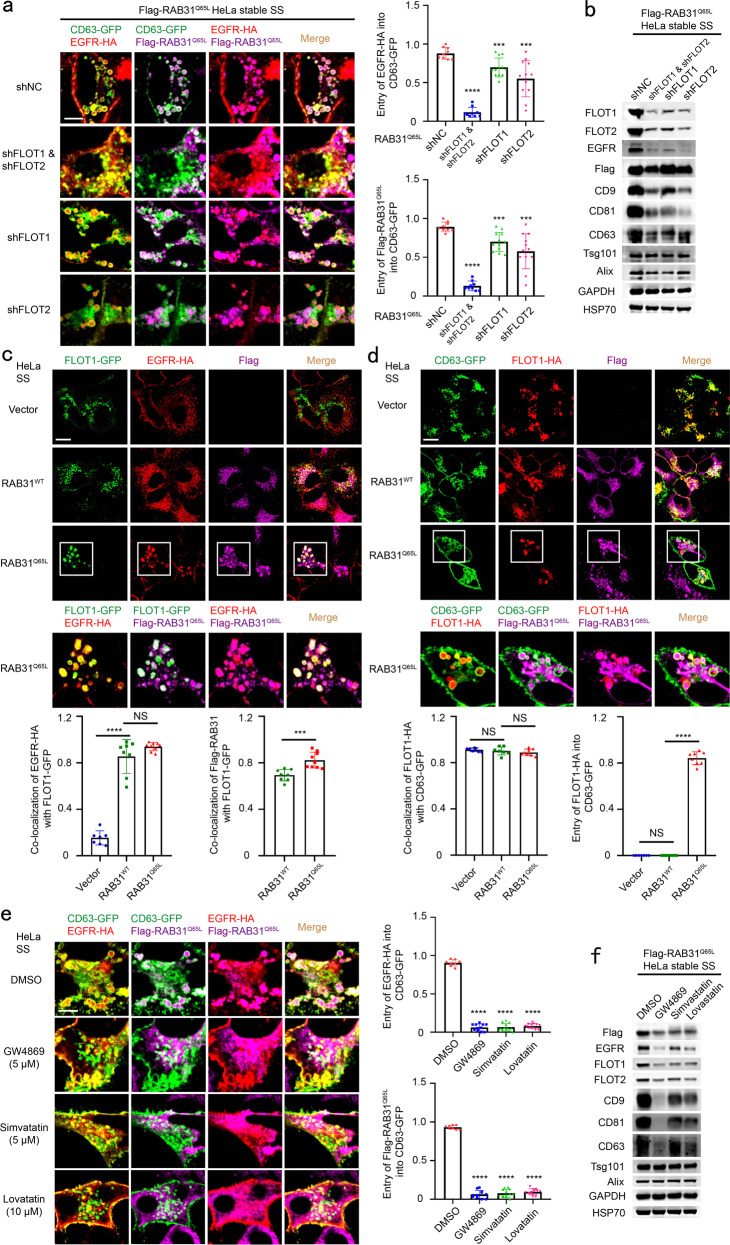
Active RAB31 engages FLOTs to drive EGFR-containing ILV formation depending on cholesterol and ceramide in lipid raft microdomains. **a** Left, immunofluorescence of EGFR-HA (red) and Flag-RAB31^Q65L^ (magenta) with CD63-GFP (green) in Flag-RAB31^Q65L^ stable HeLa cells stably expressing shNC (negative control), shFLOT1, shFLOT2 or shFLOT1 and shFLOT2 and transiently expressing EGFR-HA and CD63-GFP under serum starvation (SS). Right up panel, the ratio of entry of EGFR-HA into CD63-GFP-positive LE/MVE in shNC (*n* = 9 fields), shFLOT1 and shFLOT2 (*n* = 9 fields), shFLOT1 (*n* = 12 fields), shFLOT2 (*n* = 12 fields). Right low panel, the ratio of entry of Flag-RAB31^Q65L^ into CD63-GFP-positive LE/MVE in shNC (*n* = 9 fields), shFLOT1 and shFLOT2 (*n* = 9 fields), shFLOT1 (*n* = 12 fields), shFLOT2 (*n* = 12 fields). **b** Western blotting analyses of the concentrated conditional media from the indicated stable HeLa cells used in **a**. **c** Up panels, immunofluorescence of EGFR-HA (red) and Flag-RAB31 (magenta) with FLOT1-GFP (green) in the indicated stable HeLa cells transiently expressing EGFR-HA and FLOT1-GFP under SS. Low panel left, the ratio of co-localization of EGFR-HA with FLOT1-GFP-positive vesicle in Vector (*n* = 7 fields), RAB31^WT^ (*n* = 8 fields) and RAB31^Q65L^ (*n* = 9 fields). Low panel right, the ratio of co-localization of Flag-RAB31 with FLOT1-GFP-positive vesicle in RAB31^WT^ (*n* = 8 fields) and RAB31^Q65L^ (*n* = 9 fields). **d** Up panels, immunofluorescence of FLOT1-HA (red) and Flag-RAB31 (magenta) with CD63-GFP (green) in the indicated stable HeLa cells transiently expressing FLOT1-HA and CD63-GFP under SS. Low panel left, the ratio of co-localization of FLOT1-HA with CD63-GFP-positive LE/MVE in Vector (*n* = 7 fields), RAB31^WT^ (*n* = 7 fields) and RAB31^Q65L^ (*n* = 8 fields). Low panel right, the ratio of entry of FLOT1-HA into CD63-GFP-positive LE/MVE in Vector (*n* = 7 fields), RAB31^WT^ (*n* = 7 fields) and RAB31^Q65L^ (*n* = 8 fields). **e** Left, immunofluorescence of EGFR-HA (red) and Flag-RAB31^Q65L^ (magenta) with CD63-GFP (green) in Flag-RAB31^Q65L^ stable HeLa cells transiently expressing EGFR-HA and CD63-GFP and treated with DMSO, 5 μM GW4869, 5 μM simvastatin or 10 μM lovastatin under SS. Right up panel, the ratio of entry of EGFR-HA into CD63-GFP-positive LE/MVE in DMSO (*n* = 8 fields), GW4869 (*n* = 11 fields), simvastatin (*n* = 13 fields) and lovastatin (*n* = 12 fields). Right low panel, the ratio of entry of Flag-RAB31^Q65L^ into CD63-GFP-positive LE/MVE in DMSO (*n* = 8 fields), GW4869 (*n* = 11 fields), simvastatin (*n* = 13 fields) and lovastatin (*n* = 12 fields). **f** Western blotting analyses of the concentrated conditional media from the indicated stable HeLa cells used in **e**. All data are means ± SD. Unpaired *t*-test was used to analyze the difference between the two groups. ****P < 0.0001, ***P < 0.001, NS, no statistical significance. Scale bars, 10 μm.

Notably, flotillin proteins (FLOTs) containing FLOT1 and FLOT2, defined as canonical EV markers^41^ and lipid rafts-associated proteins in endosomes and cell membrane,^1,10,40,42,43^ were highly enriched in the concentrated conditional media driven by RAB31^Q65L^ (Fig. 2h). It has been shown that FLOTs play crucial roles in the regulation of clathrin-independent endocytosis^44^ and that the coassembly of FLOTs into microdomains induces plasma membrane curvature, budding, and accumulation of intracellular vesicles.^45^ Therefore, we sought to test whether FLOTs are the effectors for this process that RAB31^Q65L^ drives EGFR budding into CD63-positive MVEs to form ILVs. Indeed, knockdown of FLOT1, FLOT2 or both dramatically decreased the entry of EGFR and RAB31^Q65L^ into CD63-positive MVEs and the production of EGFR-containing exosomes (Fig. 3a, b; Supplementary information, Fig. S5a), suggesting that both FLOT1 and FLOT2 are required for the membrane budding of MVEs to form ILVs driven by active RAB31. In addition, western blotting analyses showed that knocking down of either FLOT1 or FLOT2 resulted in the decreases of FLOTs (Supplementary information, Fig. S5a). Since FLOT1 and FLOT2 can form homodimer and heterodimer,^43^ depletion of either one may influence the stability of another protein. Consistently, both RAB31^Q65L^ and EGFR were perfectly co-localized with FLOT1-GFP or FLOT2-GFP in MVEs (Fig. 3c; Supplementary information, Fig. S5b), and RAB31^Q65L^ drove FLOT1 or FLOT2 entry into CD63-positive MVEs to form ILVs (Fig. 3d; Supplementary information, Fig. S5c–i). In essence, exosomes are lipid rafts vesicles enriched with certain proteins, cholesterol and sphingolipid, which includes ceramide, sphingomyelin, glycosphingolipid and ganglioside, etc., depending on the parental cell types.^14,46–52^ Remarkably, disruption of lipid raft microdomains, by decreasing either ceramide (the central molecule in sphingolipid metabolism^51,52^) via inhibiting neutral sphingomyelinase using GW4869 or cholesterol via impeding 3-hydroxy-3-methyl glutaryl coenzyme A reductase using simvastatin or lovastatin, also resulted in similar results to those of FLOTs knockdown (Fig. 3e, f), suggesting that cholesterol and ceramide are required for the membrane budding of MVEs induced by FLOTs. In addition, both Tsg101 and Alix were not changed in the concentrated conditional media under treatment with DMSO, GW4869, simvastatin or lovastatin (Fig. 3f), suggesting that cholesterol and ceramide are not required for the biogenesis of exosomes driven by ESCRT. Collectively, these results demonstrate that RAB31^Q65L^ engages FLOTs to drive EGFR-containing ILV formation depending on cholesterol and ceramide in lipid raft microdomains.

### Active RAB31 interacts with the SPFH domain and drives ILV formation via the flotillin domain of FLOTs

**Fig. 4:**
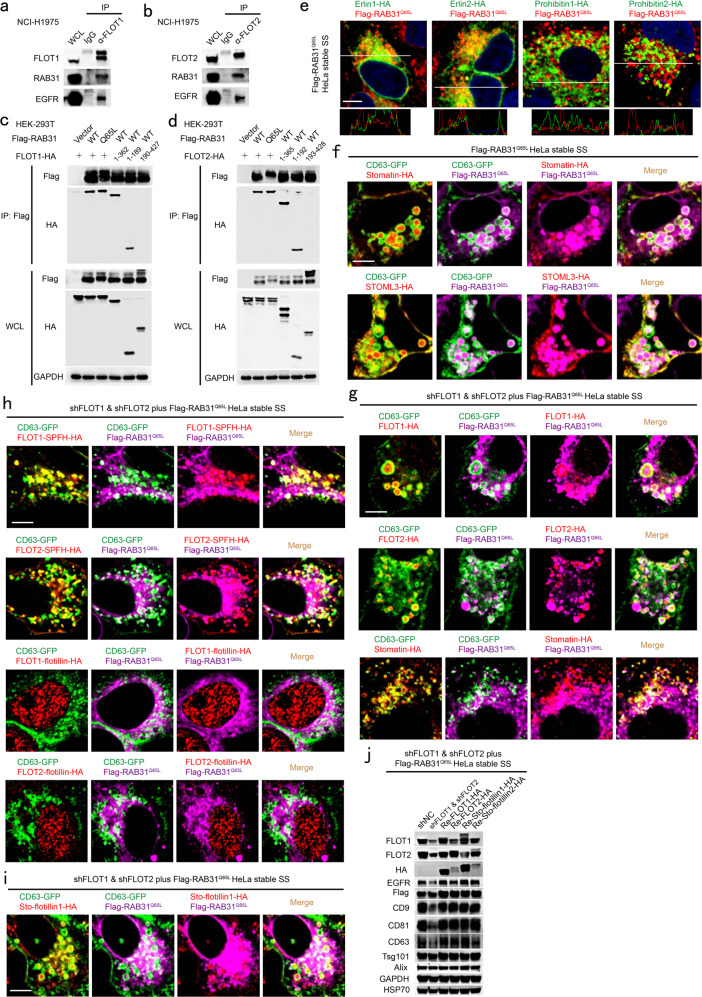
Active RAB31 interacts with the SPFH domain of FLOTs and drives MVE membrane budding to form ILVs via the Flotillin domain of FLOTs. **a**, **b** Western blotting analyses of whole-cell lysates (WCLs) and immunoprecipitates (IP) at their endogenous levels from NCI-H1975 cells using anti-FLOT1 antibody (**a**) or anti-FLOT2 antibody (**b**). **c**, **d** Western blotting analyses of WCL and IP from HEK-293T cells co-expressing the indicated plasmids. **e** Immunofluorescence of erlin1-HA, erlin2-HA, prohibitin1-HA or prohibitin2-HA (green) with Flag-RAB31^Q65L^ (red) in HeLa cells stably expression Flag-RAB31^Q65L^ and transiently expressing the indicated plasmids under serum starvation (SS). **f** Immunofluorescence of stomatin-HA or STOML3-HA (red) and Flag-RAB31^Q65L^ (magenta) with CD63-GFP (green) in HeLa cells stably expression Flag-RAB31^Q65L^ and transiently expressing the indicated plasmids under SS. **g** Immunofluorescence of FLOT1-HA, FLOT2-HA or stomatin-HA and Flag-RAB31^Q65L^ (magenta) with CD63-GFP (green) in the indicated stable HeLa cells transiently expressing the indicated plasmids under SS. **h** Immunofluorescence of FLOT1-SPFH-HA, FLOT2-SPFH-HA, FLOT1-flotillin-HA, or FLOT2-flotillin-HA (red) and Flag-RAB31^Q65L^ (magenta) with CD63-GFP (green) in the indicated stable HeLa cells transiently expressing the indicated plasmids under SS. **i** Immunofluorescence of Sto-flotillin1-HA (red) chimeras and Flag-RAB31^Q65L^ (magenta) with CD63-GFP (green) in the indicated stable HeLa cells transiently expressing the indicated plasmids under SS. **j** Western blotting analyses of the concentrated conditional media from the indicated stable HeLa cells stably re-introduced with the indicated plasmids. Scale bars, 10 μm.

We further investigated the mechanism underlying ILV formation driven by the RAB31^Q65L^/FLOTs machinery. The endogenous and exogenous interaction between RAB31 and FLOTs were detected (Fig. 4a, b; Supplementary information, Fig. S6a, b), and FLOTs could simultaneously immunoprecipitate RAB31 and EGFR at their endogenous levels in cells (Fig. 4a, b), and each SPFH domain of FLOTs, rather than the flotillin domain, was responsible for such an interaction (Fig. 4c, d). FLOTs belong to the SPFH (stomatin/prohibitin/flotillin/HflK/C) domain-containing protein family that are localized in the lipid raft microdomains in diverse cellular membranes.^42,43^ Indeed, proteins containing the SPFH domain, such as erlin1, erlin2, prohibitin1, prohibitin2, stomatin and stomatin-like protein 3 (STOML3), could also be immunoprecipitated by RAB31 (Supplementary information, Fig. S6c), suggesting that the SPFH domain is the common and intrinsic structure pattern interacting with RAB31. However, erlins (erlin1 and erlin2) and prohibitins (prohibitin1 and prohibitin2), identified as lipid rafts markers for endoplasmic reticulum and mitochondria,^42^ respectively, had little correlation with the distribution of RAB31^Q65L^ on ILVs (Fig. 4e). Consistent with the localization of both stomatin and STOML3 in the lipid rafts of endosomes, they entered CD63-positive MVEs (Fig. 4f). As expected, either FLOT1 or FLOT2, but not stomatin, could rescue the phenotype of FLOTs knockdown (Fig. 4g). Notably, although stomatin, FLOT1-SPFH and FLOT2-SPFH, lacking of the flotillin domain, could also localize to CD63-positive late endosomes, and FLOT1-flotillin or FLOT2-flotillin were distributed as aggregates in the nucleus, neither of them could rescue the phenotype of FLOTs knockdown (Fig. 4g, h). Together, these results reveal that the SPFH domain of FLOTs is responsible for the interaction with RAB31 and that the flotillin domain of FLOTs is responsible for the membrane budding of CD63-positive MVEs driven by RAB31^Q65L^. To further validate this conclusion, we generated the chimeras Sto-flotillin1 and Sto-flotillin2, which contain the N-terminal SPFH domain from stomatin conjugated with the flotillin domain from FLOT1 and FLOT2, respectively. Indeed, RAB31^Q65L^ drove Sto-flotillin1 and Sto-flotillin2 to enter CD63-positive MVEs (Fig. 4i; Supplementary information, Fig. S6d), and Sto-flotillin1 and Sto-flotillin2 could completely rescue the production of EGFR-containing exosomes in cells with knockdown of FLOTs (Fig. 4j; Supplementary information, Fig. S6e).

Next, we further deciphered the minimal residues of FLOTs responsible for their interaction with RAB31. As illustrated in Supplementary information, Fig. S6f, the ^78^KEML^81^ or ^88^FLGK^91^ motif in alpha helix 1 (AH1) of FLOT1 and both ^96^VQDI^99^ and ^104^LQTL^107^ motifs in AH2 of FLOT2 were responsible for the interactions of FLOT1 and FLOT2 with RAB31^Q65L^, respectively (Supplementary information, Fig. S6g, h). Interestingly, these deletions or mutants of FLOTs (FLOT1ΔAH1, FLOT1/AH1/M1, FLOT2ΔAH2, FLOT2/AH2/M4) did not localize to CD63 positive late endosomes even in the presence of endogenous FLOTs (Supplementary information, Fig. S6i). As expected, RAB31^Q65L^ could not drive these deletions or mutants of FLOTs to enter CD63-positive MVEs in FLOTs-knockdown cells (Supplementary information, Fig. S6j). Together, these results elucidate that the AH1 of FLOT1 and AH2 of FLOT2 are not only required for their correct localization but also required for the interaction with RAB31^Q65L^, which is responsible for the membrane budding of CD63-positive MVEs driven by RAB31^Q65L^.

### Tyrosine phosphorylation of RAB31 by active EGFR acts similarly to its active form

**Fig. 5:**
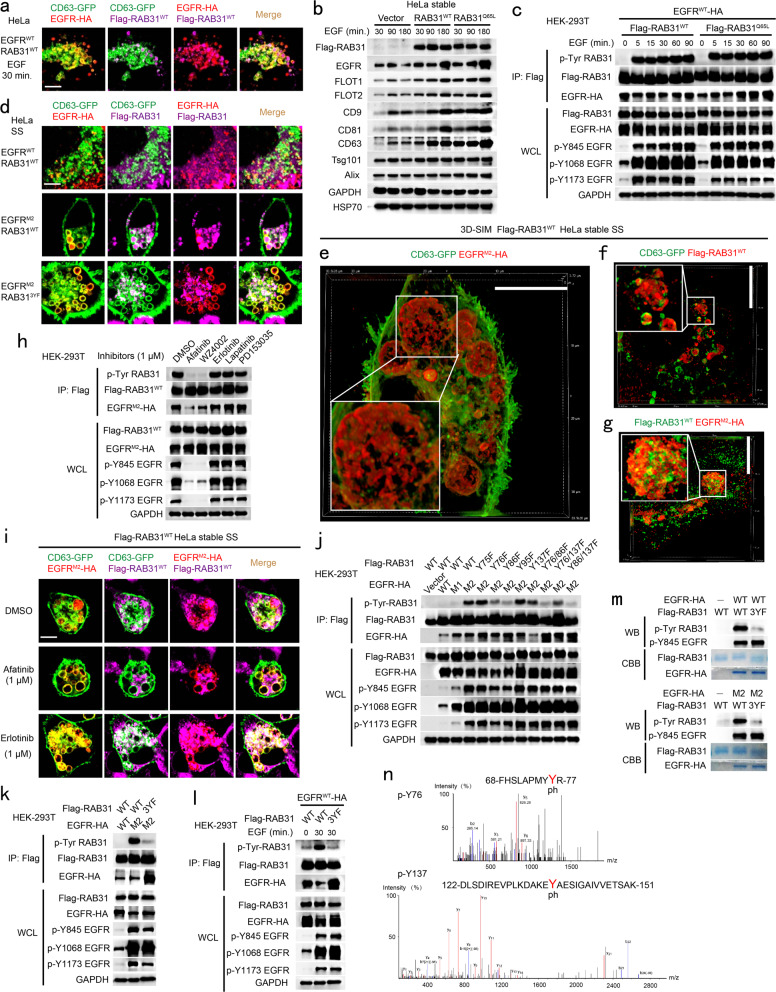
Tyrosine phosphorylation of RAB31 by active EGFR acts similarly to its active form. **a** Immunofluorescence of EGFR-HA (red) and Flag-RAB31^WT^ (magenta) with CD63-GFP (green) in Flag-RAB31^WT^ stable HeLa cells transiently expressing EGFR-HA and CD63-GFP stimulated with EGF for 30 min as indicated. **b** Western blotting analyses of the concentrated conditional media from the indicated stable HeLa cells treated with 100 ng/mL of EGF at the indicated times. **c** Western blotting analyses of whole-cell lysates (WCL) and immunoprecipitates (IP) from HEK-293T cells co-expressing the indicated plasmids treated with 100 ng/mL of EGF at the indicated times. p-Tyr, anti-phosphotyrosine antibody. **d** Immunofluorescence of EGFR-HA (red) and Flag-RAB31 (magenta) with CD63-GFP (green) in the indicated Flag-RAB31 stable HeLa cells transiently expressing EGFR-HA and CD63-GFP under serum starvation (SS). **e**–**g** Immunofluorescence of EGFR^M2^-HA (red) with CD63-GFP (green) (**e**), Flag-RAB31^WT^ (red) with CD63-GFP (green) (**f**) and Flag-RAB31^WT^ (green) with EGFR^M2^-HA (red) (**g**) in Flag-RAB31^WT^ stable HeLa cells transiently expressing EGFR^M2^-HA and CD63-GFP under SS using 3D-SIM. **h** Western blotting analyses of WCL and IP from HEK-293T cells co-expressing EGFR^M2^-HA and Flag-RAB31^WT^ treated with the indicated EGFR-tyrosine kinase inhibitors under SS. **i** Immunofluorescence of EGFR^M2^-HA (red) and Flag-RAB31^WT^ (magenta) with CD63-GFP (green) in Flag-RAB31^WT^ stable HeLa cells transiently expressing EGFR^M2^-HA and CD63-GFP treated with the indicated inhibitors under SS. **j**, **k** Western blotting analyses of WCL and IP from HEK-293T cells co-expressing the indicated plasmids under SS. **l** Western blotting analyses of WCL and IP from HEK-293T cells co-expressing the indicated plasmids treated with EGF for the indicated times. **m** Western blotting (WB) and Coomassie brilliant blue (CBB) analyses of the purified different EGFR and RAB31 forms as indicated after in vitro kinase assay, as described in Materials and Methods section. **n** Mass spectrometry analysis of the phosphorylated tyrosine sites in RAB31 purified from in vitro kinase assay. Scale bars, 10 μm.

RAB31^WT^ only directs EGFR to the membrane of CD63-positive late endosomes in HeLa cells under serum starvation (Fig. 2a, f). Interestingly, activation of EGFR by EGF for 30 min drove both RAB31^WT^ and EGFR entry into CD63-positive MVEs to form ILVs and produce more EGFR-containing exosomes during EGF treatment (Fig. 5a, b; Supplementary information, Fig. S7a–c), indicating that RAB31^WT^ has similar functions to RAB31^Q65L^ in cells upon EGF stimulation. Therefore, we hypothesized that active EGFR probably phosphorylates RAB31, which in turn activates RAB31. Indeed, activation of EGFR by EGF resulted in tyrosine phosphorylation of both RAB31^WT^ and RAB31^Q65L^ (Fig. 5c). Many somatic mutations of EGFR, such as L858R (M1), L858R/T790M (M2), L858R/T790M/C797S (M3), Del E746-A750 (D1), Del E746-A750/T790M (D2) and Del E746-A750/T790M/C797S (D3), have been proven to be active without ligands in non-small cell lung cancer (NSCLC).^28,29,53,54^ These active EGFR mutants, but not WT EGFR (EGFR^WT^) resulted in tyrosine phosphorylation of both RAB31^WT^ and RAB31^Q65L^ (Supplementary information, Fig. S7d), and also drove RAB31^WT^ and themselves entry into CD63-positive MVEs to form ILVs under serum starvation (Fig. 5d–g; Supplementary information, Fig. S7e, f). Furthermore, the EGFR inhibitors, Afatinib and WZ4002 (inhibit the activation of EGFR T790M mutants, activated mutants and WT), but not Erlotinib (inhibit the activation of EGFR activated mutants and WT), Lapatinib or PD153035 (inhibit the activation of EGFR-WT), abrogated tyrosine phosphorylation of both EGFR^M2^ and RAB31 (Fig. 5h). Additionally, Afatinib, but not Erlotinib, dramatically diminished both EGFR^M2^ and RAB31^WT^ entry into CD63-positive MVEs, as well as the production of EGFR^M2^-containing exosomes (Fig. 5i; Supplementary information, Fig. S7g, h).

To identify which tyrosine residue(s) of RAB31 were phosphorylated by active EGFR, multiple RAB31 mutants were generated with single, double, or triple tyrosines (Y) mutated into phenylalanines (F). We found that Y76, Y86 and Y137 as dominant phosphorylation sites were phosphorylated by active EGFR in cells, such as those expressing EGFR^M2^ or EGFR^WT^ upon EGF stimulation (Fig. 5j–l), and the non-tyrosine-phosphorylation form of RAB31, RAB31^3YF^ (Y76F, Y86F and Y137F), could not drive EGFR^M2^ entry into CD63-positive MVEs (Fig. 5d) and markedly decreased the production of EGFR^M2^-containing exosomes (Supplementary information, Fig. S7i, j), indicating that tyrosine phosphorylation of RAB31 by EGFR^M2^ is required to drive EGFR^M2^ entry into CD63-positive MVEs to produce EGFR^M2^-containing exosomes. Interestingly, any single tyrosine phosphorylation of RAB31 at Y76, Y86 or Y137 was sufficient to drive EGFR^M2^ entry into CD63-positive MVEs (Supplementary information, Fig. S7k). Furthermore, in vitro kinase assay showed that RAB31^WT^, but not RAB31^3YF^, was directly phosphorylated by both EGFR^WT^ and EGFR^M2^ (Fig. 5m). Additionally, mass spectrometry analysis showed that Y76 and Y137 were identified to be the phosphorylated sites in RAB31 purified from in vitro kinase assay (Fig. 5n). Together, these results suggest that active EGFR can switch RAB31 to be active via tyrosine phosphorylation in cells.

### RTKs phosphorylate RAB31 to drive them entry into CD63-positive MVEs

Because HER2, IGF1R, MET and NTRK2 are also present in tumor-derived exosomes that play important roles in tumor progression,^55–58^ we aimed to investigate whether RAB31 control these RTKs entry into CD63-positive MVEs. Very interestingly, RAB31^Q65L^ drove HER2, IGF1R, MET, NTRK2, PDGFR-α, PDGFR-β, FGFR1, or FGFR2 entry into CD63-positive MVEs to probably form ILVs according to the results of EGFR (Supplementary information, Fig. S8a), indicating that active RAB31 may generally control these RTKs ILV formation.

EGFR enables RAB31 to be active via tyrosine phosphorylation, and active RAB31 engages flotillin proteins in lipid raft microdomains to drive EGFR entry into MVEs to form ILVs. We further investigated whether RAB31 employs the similar mechanism to drive HER2, IGF1R, MET, NTRK2, PDGFR-α, PDGFR-β, FGFR1, or FGFR2 entry into CD63-positive MVEs to form ILVs. Indeed, these RTK members were immunoprecipitated by FLOT1 or FLOT2 (Supplementary information, Fig. S8b, c), suggesting that these RTK members can be distributed in the FLOTs-associated lipid raft microdomains. Consistently, activation of IGF1R, MET, PDGFR-β, or FGFR2 by their corresponding ligands also drove RAB31^WT^ and themselves to enter CD63-positive MVEs to form ILVs (Supplementary information, Fig. S8d–g), and these active RTKs could phosphorylate RAB31^WT^ (Supplementary information, Fig. S8h–k). Together, these results reveal a common mechanism that RTKs phosphorylate RAB31 to switch RAB31 to the active form that engages flotillin proteins in lipid raft microdomains to drive these RTKs entry into MVEs to form ILVs.

**Fig. 6:**
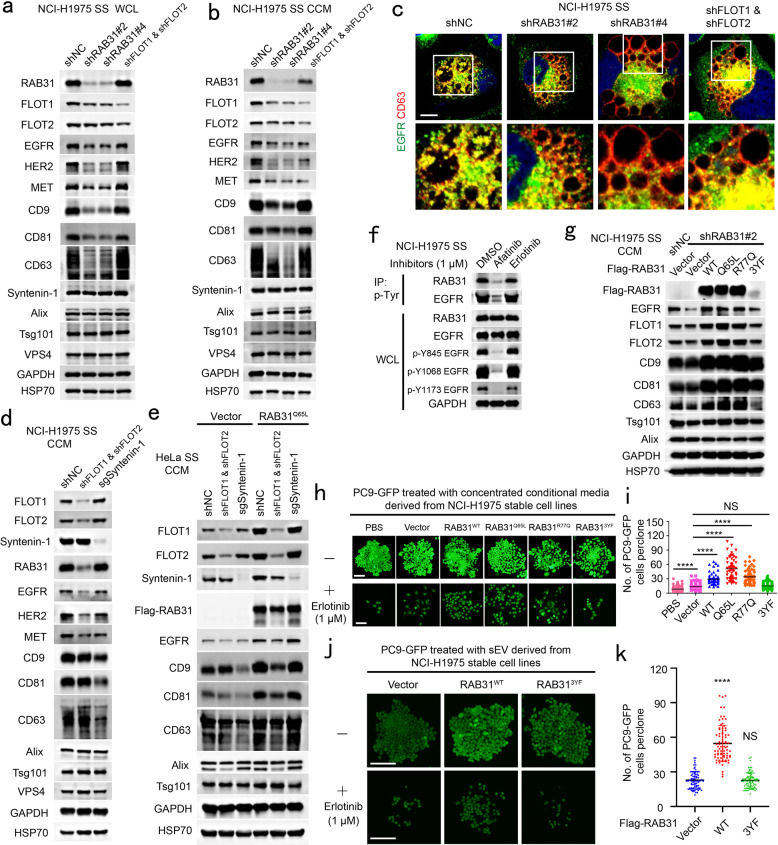
EGFR phosphorylates RAB31 to drive EGFR into exosomes and the exosomes promoted by RAB31 mediate resistance to erlotinib. **a** Western blotting analyses of whole-cell lysates (WCL) from the indicated NCI-H1975 cells stably expressing shNC, shRAB31 or shFLOT1 and shFLOT2. **b** Western blotting analyses of the concentrated conditional media from the indicated stable NCI-H1975 cells used in **a** under serum starvation (SS). **c** Immunofluorescence of endogenous EGFR (green) and CD63 (red) in the indicated stable NCI-H1975 cells used in **a** under SS. **d** Western blotting analyses of the concentrated conditional media from the indicated stable NCI-H1975 cells under serum starvation (SS). **e** Western blotting analyses of the concentrated conditional media from the indicated stable HeLa cells under serum starvation (SS). **f** Western blotting analyses of WCL and immunoprecipitates (IP) from NCI-H1975 cells treated with afatinib or erlotinib under SS. **g** Western blotting analyses of the concentrated conditional media from the indicated stable NCI-H1975 cells under SS. **h** Representative clone images of PC9-GFP cells treated with the concentrated conditional media derived from the indicated stable NCI-H1975 cells without or with erlotinib. **i** Quantification of the numbers of each clone for **h**. Data are means ± SD of cell numbers in each clone with PBS (*n* = 75), Vector (*n* = 82), RAB31^WT^ (*n* = 62), RAB31^Q65L^ (*n* = 62), RAB31^R77Q^ (*n* = 65) or RAB31^3YF^ (*n* = 84). **j** Representative clone images of PC9-GFP cells treated with the pure small EV (sEV) derived from the indicated stable NCI-H1975 cells without or with erlotinib. **k** Quantification of the numbers of each clone for **j**. Data are means ± SD of cell numbers in each clone with Vector (*n* = 71), RAB31^WT^ (*n* = 80) or RAB31^3YF^ (*n* = 80). Unpaired *t*-test was used to analyze the difference between the two groups. ****P < 0.0001, NS, no statistical significance. Scale bars, 10 μm (**c**) and 100 μm (**h** and **j**).

To further validate whether endogenous RAB31 and FLOTs were required for RTKs exosomes, endogenous RAB31 or FLOTs were knocked down in NCI-H1975 cells harboring endogenous EGFR^M2^. Knockdown of RAB31 resulted in the decrease of CD9, CD81, CD63 and other membrane proteins tested in the concentrated conditional media mainly owing to their protein decreases in cells (Fig. 6a, b). Knockdown of FLOTs resulted in no effect of CD9, CD81, CD63 and other membrane proteins in cells, but significantly decreased exosomal RAB31, EGFR, HER2 and MET protein levels (Fig. 6a, b). Expectedly, depletion of RAB31 or FLOTs substantially diminished the entry of endogenous EGFR^M2^ into CD63-positive MVEs (Fig. 6c). Together, these results suggest that the RAB31-FLOTs machinery marks a lipid raft microdomains-dependent exosome pathway that controls RTKs sorting into exosomes. In addition, CD9, CD81, CD63, Syntenin-1 and Alix in the concentrated conditional media from FLOTs-depleted NCI-H1975 or HeLa cells were not changed (Fig. 6b, d, e; Supplementary information, Fig. S9a, b). As expected, depletion of Syntenin-1 significantly decreased CD9, CD81 and CD63 in the concentrated conditional media from NCI-H1975 or HeLa cells (Fig. 6d, e; Supplementary information, Fig. S9a, b), which was consistent with the notion that these tetraspanins are sorted into exosomes through the Syntenin-Alix-ESCRT-III pathway.^1,10,13^ Whereas RAB31, FLOTs, EGFR, HER2 and MET in the concentrated conditional media from the Syntenin-1-depleted NCI-H1975 cells were not changed (Fig. 6d), indicating that RAB31-FLOTs and Syntenin-Alix-ESCRT-III machineries are two parallel exosome pathways, which are responsible for different cargoes. Interestingly, RAB31^Q65L^ promoted CD9, CD81, CD63 and Syntenin-1, but not Alix, in the concentrated conditional media derived from HeLa cells (Fig. 6e), and knockdown of FLOTs reduced CD9, CD81, CD63 and Syntenin-1 in the concentrated conditional media derived from RAB31^Q65L^-stable HeLa cells to the similar level compared with the Vector group (Fig. 6e), suggesting that the elevated secretion of these proteins promoted by RAB31^Q65L^ is dependent on FLOTs, and that Syntenin-1 can be switched from a driver to cargo owing to the binding to the cytoplasmic tails of these tetraspanins^12^ when RAB31^Q65L^ is overexpressed. Unexpectedly, depletion of Syntenin-1 did not reduce CD9, CD81 and CD63 in the concentrated conditional media when RAB31^Q65L^ is present (Fig. 6e), suggesting that these tetraspanins can be hijacked by active RAB31-FLOTs machinery. Moreover, previous study has shown that overexpression of Syntenin does not increase the protein level of exosomal FLOT1.^10^ Therefore, we speculate that Syntenin-Alix-ESCRT-III pathway is required for these tetraspanins sorting into exosomes as a basal constitutive secretion, whereas active RAB31-FLOTs machinery strongly triggered by upstream signals (e.g., EGFR) can drive these tetraspanins and their binding partner Syntenin-1 sorting into exosomes bypassing the Alix-ESCRT-III pathway.

We further showed that inhibition of endogenous EGFR^M2^ by Afatinib, but not by Erlotinib, dramatically decreased tyrosine phosphorylation of both endogenous EGFR^M2^ and RAB31, as well as that of both exogenous RAB31^WT^ and RAB31^Q65L^ (Fig. 6f; Supplementary information, Fig. S9c). Afatinib, but not by Erlotinib, also significantly impaired the entry of endogenous EGFR^M2^ and RAB31 into CD63-positive MVEs (Supplementary information, Fig. S9d, e). More importantly, RAB31^WT^, RAB31^Q65L^ and RAB31^R77Q^ (the mutant that functions similarly to RAB31^Q65L^ in NSCLC), but not RAB31^3YF^, could rescue the phenotype to promote endogenous EGFR^M2^, FLOT1, FLOT2, CD9, CD81 and CD63 in the concentrated conditional media (Fig. 6g), whereas all of these RAB31 forms including RAB31^3YF^ could rescue the decrease of these membrane proteins in cells with stable knockdown of endogenous RAB31 (Supplementary information, Fig. S9f). These results suggest that the phosphorylation of RAB31 mediated by EGFR plays a crucial role in the production of EGFR^M2^-containing exosomes.

### Active RAB31 promotes endogenous EGFR^M2^-containing exosomes mediating resistance to erlotinib

Tumor-derived EGFR-containing exosomes have been shown to be crucial for the tumor microenvironment, cancer cell proliferation and metastasis, as well as for suppressing host innate immunity.^30,32–34^ In addition, an increasing number of studies have indicated that exosomes are involved in drug resistance in various cancers,^59^ and that NCI-H1975 cells harboring EGFR^M2^ and PC9 cells harboring EGFR^D1^ are resistant and sensitive to Erlotinib, respectively. We asked whether the EGFR^M2^-containing exosomes from donor cells confer drug resistance to their recipient cells using the co-culture of PC9-GFP cells with NCI-H1975 cells stably expressing vector, RAB31^Q65L^, RAB31^R77Q^, RAB31^WT^ or RAB31^3YF^ (Supplementary information, Fig. S9g). After co-culture for 10 days, when the densities of PC9-GFP cells among these five groups were similar, the PC9-GFP cells were treated with Erlotinib for 5 days. Their cell numbers were in the sequential order of RAB31^Q65L^, RAB31^R77Q^, RAB31^WT^ and RAB31^3YF^ from the highest to the lowest ones (Supplementary information, Fig. S9i, j). This sequential order of RAB31 forms is the same as that of their ability to drive the production of endogenous EGFR^M2^-containing exosomes (Supplementary information, Fig. S9h), and the RAB31^3YF^ group was almost similar to the Vector group. To further validate that the resistance of PC9-GFP cells to erlotinib was mediated by small EVs derived from NCI-H1975 cells, PBS or the concentrated conditional media were obtained from Flag-RAB31 NCI-H1975 stable cells and were added into the media for PC9-GFP cells. After 9 days, when the densities of PC9-GFP cells among these six groups were similar (Fig. 6h), the PC9-GFP cells were treated with Erlotinib for 6 days. Similar to the results of co-culture assay, RAB31^Q65L^, RAB31^R77Q^, RAB31^WT^, but not RAB31^3YF^, significantly promoted the number of survival PC9-GFP cells compared with Vector group (Fig. 6h, i). Notably, Vector NCI-H1975 group also significantly promoted the number of survival PC9-GFP cells compared with PBS group (Fig. 6h, i). To further exclude the effect of NV material co-concentrated with small EVs, we used density gradient fractionation to isolate pure small EVs from the concentrated conditional media (Supplementary information, Fig. S9k). The pure small EVs were added into the media for PC9-GFP cells for 9 days, and then the PC9-GFP cells were treated with Erlotinib for 6 days. Similar to the results of concentrated conditional media treatment, RAB31^WT^, but not RAB31^3YF^, significantly promoted the number of survival PC9-GFP cells compared with the Vector group (Fig. 6j, k). Together, these results demonstrate that the endogenous EGFR^M2^-containing exosomes driven by RAB31 from NCI-H1975 donor cells render their recipient PC9-GFP cells to become resistant to Erlotinib.

### RAB31 sequesters EGFR in CD63-positive MVEs to prevent its lysosomal degradation

**Fig. 7:**
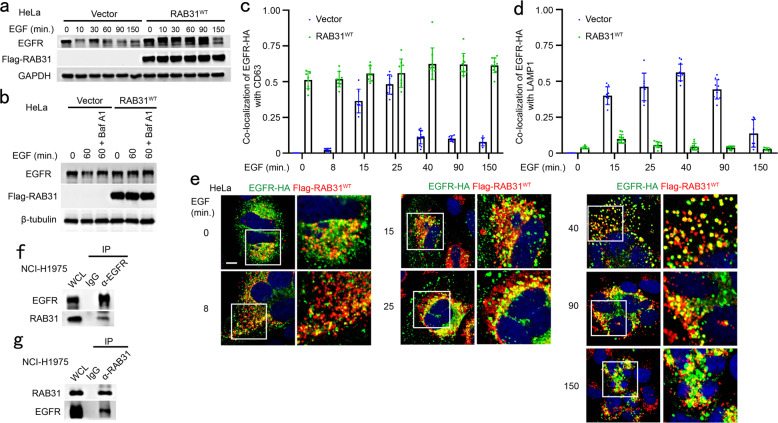
RAB31 sequesters EGFR in CD63-positive MVEs to prevent its lysosomal degradation. **a** Western blotting analyses of whole-cell lysates (WCL) from the indicated stable HeLa cells treated with 100 ng/mL of EGF at the indicated time points. **b** Western blotting analyses of WCL from the indicated stable HeLa cells treated with 100 ng/mL of EGF or pre-treated with Bafilomycin A1 (Baf A1) for 6 h and then treated with 100 ng/mL of EGF at the indicated time points. **c** The ratio of co-localization of EGFR-HA with CD63-positive LE/MVE in Vector and Flag-RAB31^WT^ stable HeLa cells treated with 100 ng/mL of EGF at the indicated times for Supplementary information, Fig. S10a. **d** The ratio of co-localization of EGFR-HA with LAMP1-positive lysosome in Vector and Flag-RAB31^WT^ stable HeLa cells treated with 100 ng/mL of EGF at the indicated times for Supplementary information, Fig. S10b. **e** Immunofluorescence of EGFR-HA (green) with Flag-RAB31^WT^ (red) in Flag-RAB31^WT^ stable HeLa cells transiently expressing EGFR-HA treated with 100 ng/mL of EGF at the indicated times. Western blotting analyses of WCL and immunoprecipitation (IP) at their endogenous levels from NCI-H1975 cells using anti-EGFR (**f**) or anti-RAB31 (**g**) antibodies. Scale bars, 10 μm.

Ectopic RAB31 retains EGFR in CD63-positive late endosomes and MVEs rather than lysosomes, and depletion of RAB31 induces the decrease of EGFR, as determined above. Indeed, by monitoring the degradation and trafficking of EGFR in cells treated with EGF, we found that RAB31 could prevent the degradation of EGFR in cells stimulated with EGF (Fig. 7a). The accumulation of EGFR in cells stably expressing RAB31 was similar to that of the inhibition of EGFR lysosomal degradation pretreated with Bafilomycin A1 in Vector cells (Fig. 7b), indicating that RAB31 prevents EGFR lysosomal degradation. EGFR was mainly localized from the early to late endosomes upon EGF stimulation for 8 to 25 min (Fig. 7c; Supplementary information, Fig. S10a). However, EGFR was still blocked in CD63-positive MVEs under EGF stimulation for 40 to 150 min in cells stably expressing RAB31 (Fig. 7c; Supplementary information, Fig. S10a), whereas EGFR escaped from CD63-positive MVEs and was transported to LAMP1-positive lysosomes for degradation upon EGF stimulation for 15 to 150 min in Vector cells (Fig. 7d; Supplementary information, Fig. S10b). Consistently, RAB31 was associated with EGFR under serum starvation and during EGF treatment (Fig. 7e); the interaction between RAB31 with EGFR was detected at endogenous and exogenous levels in cells (Fig. 7f, g; Supplementary information, Fig. S10c, d). Notably, both total and the phosphorylated endogenous EGFR, but not its downstream p-AKT and p-ERK, were slightly increased by ectopic RAB31 in various cancer cell lines (Supplementary information, Fig. S10e), indicating that EGFR signaling from plasma membrane to endosomal surface^20^ is not affected by RAB31, but the activated EGFR is retained in the MVEs by RAB31. Together, these results indicate that RAB31 sequesters EGFR in CD63-positive MVEs by preventing the fusion of MVEs with lysosomes.

### RAB31 recruits TBC1D2B to inactivate RAB7

**Fig. 8:**
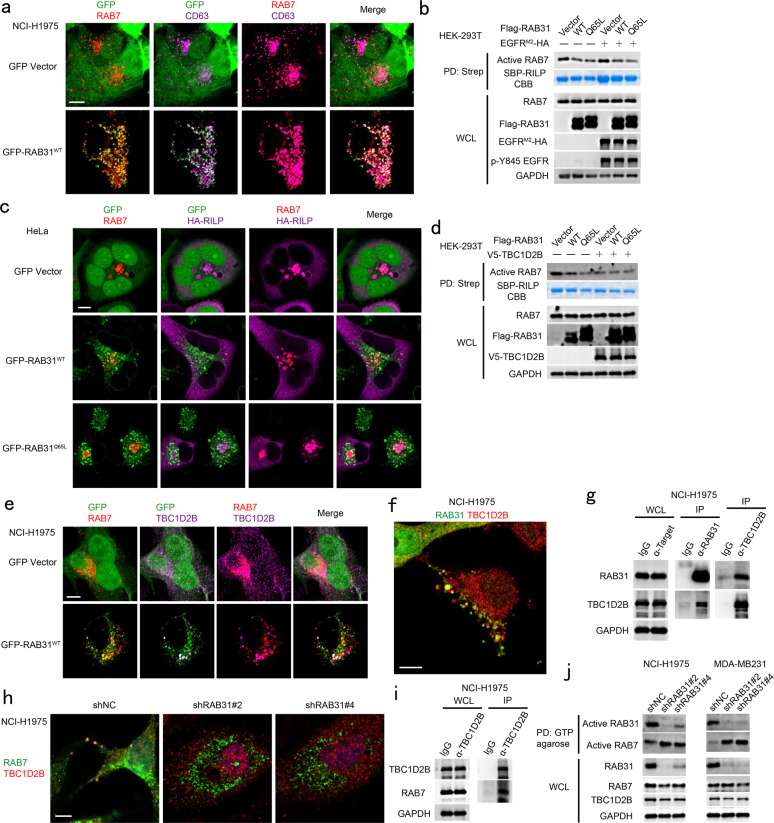
RAB31 recruits TBC1D2B to inactivate RAB7 suppressing the fusion of MVEs with lysosomes. **a** Immunofluorescence of endogenous RAB7 (red) and endogenous CD63 (magenta) with GFP-RAB31^WT^ (green) in the indicated stable NCI-H1975 cells. **b** Western blotting analyses of whole-cell lysates (WCL) and streptavidin pull-down (PD) from HEK-293T cells co-expressing the indicated plasmids with SBP-RILP. Coomassie brilliant blue (CBB) analyses of the PD of SBP-RILP. **c** Immunofluorescence of endogenous RAB7 (red) and HA-RILP (magenta) with GFP-RAB31 (green) in the indicated stable HeLa cells transiently expressing HA-RILP. **d** Western blotting analyses of WCL and streptavidin PD from HEK-293T cells co-expressing the indicated plasmids with SBP-RILP. Coomassie brilliant blue (CBB) analyses of the PD of SBP-RILP. **e** Immunofluorescence of endogenous RAB7 (red) and endogenous TBC1D2B (magenta) with GFP-RAB31 (green) in the indicated stable NCI-H1975 cells. **f** Immunofluorescence of endogenous TBC1D2B (red) with endogenous RAB31 (green) in NCI-H1975 cells. **g** Western blotting analyses of WCL and IP using the indicated antibody at their endogenous levels from NCI-H1975 cells. **h** Immunofluorescence of endogenous TBC1D2B (red) with endogenous RAB7 (green) in NCI-H1975 cells stably expressing shNC or shRAB31. **i** Western blotting analyses of WCL and IP using the indicated antibody at their endogenous levels from NCI-H1975 cells. **j** Western blotting analyses of WCL and GTP agarose PD at their endogenous levels from NCI-H1975 and MDA-MB231 cells stably expressing shNC or shRAB31. Scale bars, 10 μm.

Because the fusion of MVEs with lysosomes is mediated by active RAB7,^24,27^ we surmised that RAB31 may inactivate RAB7 to prevent the fusion of MVEs with lysosomes. Indeed, RAB31 and RAB7 could be simultaneously distributed on CD63-positive late endosomes (Fig. 8a), and the active RAB7 was decreased by ectopic RAB31 regardess of whether active EGFR is present or absent (Fig. 8b). Moreover, RAB-interacting lysosomal protein (RILP)^60^ was recruited to the active RAB7-positive lysosomes but not to the RAB31-positive late endosomes and MVEs (Fig. 8c), indicating the inactivation of RAB7 on late endosomes where high RAB31 is distributed. Notably, RAB31 specifically recruited TBC1D2B,^61^ but not other RAB7 GTPase-activating proteins (GAPs),^62^ such as TBC1D2A,^63^ TBC1D5^64^ and TBC1D15,^65^ to inactivate RAB7 on the RAB31-positive late endosomes (Supplementary information, Fig. S11a). Although TBC1D5 and RAB31 were partially co-distributed on late endosomes, TBC1D2B had much higher binding affinity to RAB31 compared to TBC1D5 (Supplementary information, Fig. S11b). However, the GAP activity of TBC1D2B towards RAB7 could not be further enhanced by RAB31 (Fig. 8d), and overexpression of RAB31 could recruit endogenous TBC1D2B to both RAB31- and RAB7-positive late endosomes (Fig. 8e). Moreover, the recruitment of TBC1D2B by RAB31, the co-localization of TBC1D2B with RAB7, and the interaction of TBC1D2B with either RAB31 or RAB7 were validated at their endogenous levels (Fig. 8f–i). The second coiled-coil domain (CC2, 393–461aa) of TBC1D2B is responsible for its recruitment by RAB31 to the RAB31-positive late endosomes (Supplementary information, Fig. S11c). Notably, depletion of RAB31 not only resulted in no distribution of endogenous TBC1D2B on the RAB7-positive late endosomes and lysosomes (Fig. 8h), but also significantly increased active RAB7 in cells (Fig. 8j). Taking together, these results reveal that RAB31 recruits TBC1D2B to inactivate RAB7, which in turn suppresses the fusion of late endosomes/MVEs with lysosomes.

## Discussion

**Fig. 9:**
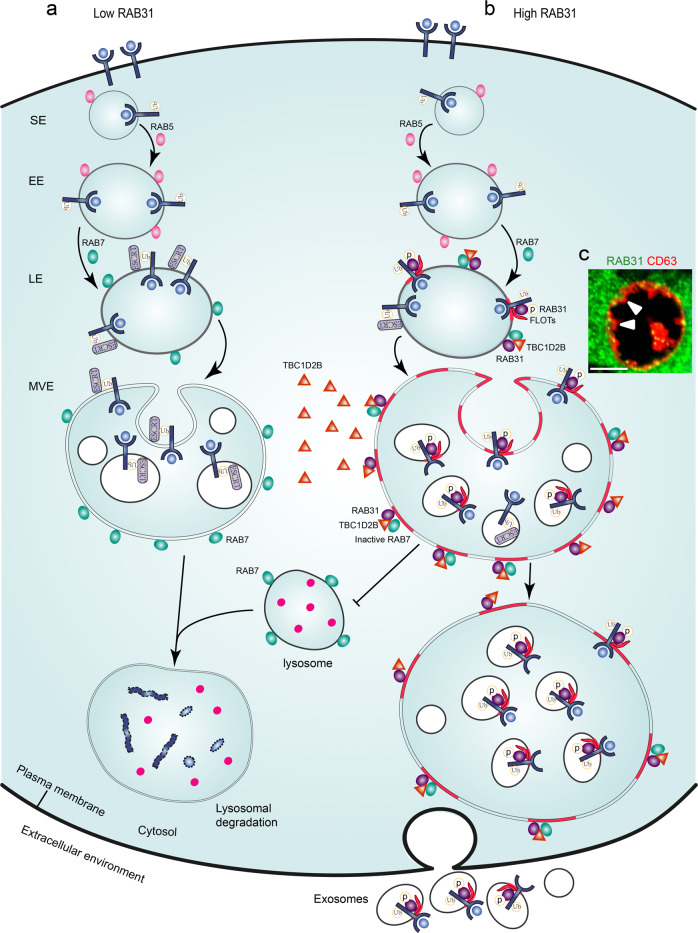
The proposed model for the functions of RAB31 in exosome pathway. EGFR are endocytosed into cells to form signaling endosomes (SE) and early endosomes (EE) regulated by RAB5, and then are transported from early to late endosomes (LE) regulated by transition from RAB5 to RAB7. **a** At this time, ESCRT machinery sorts the ubiquitylated EGFR into intraluminal vesicles (ILVs) that are destined to lysosomes for degradation by the fusion of multivesicular endosomes (MVEs) with lysosomes regulated by RAB7. **b** However, high RAB31, guarding on the late endosomes, encounters active EGFR and can be activated via tyrosine phosphorylation by EGFR, and then active RAB31 engages FLOTs in lipid rafts to drive EGFR entry into MVEs to form ILVs. Meanwhile, RAB31 recruits TBC1D2B to inactivate RAB7 preventing the fusion of MVEs with lysosomes, thereby enabling that the sequestered EGFR ILVs are secreted as exosomes. **c** Representative image of MVE membrane budding to form ILVs driven by the active RAB31 in NCI-H1975 cells. The white triangles indicate the budding moments of MVE membrane. Immunofluorescence of endogenous RAB31 (green) and CD63 (red) in NCI-H1975 cells. Scale bar, 5 μm.

Our findings reveal the critical roles of RAB31 in EGFR fates and define RAB31 not only as a driver for ILV formation but also as a key balance factor avoiding endolysosomal degradation for exosome biogenesis. Low level of RAB31 does not prevent the degradation of activated EGFR from cytomembrane to lysosomes (Fig. 9a). High level of RAB31, guarding on the late endosomes, encounters active EGFR and can be activated via tyrosine phosphorylation by EGFR, and then active RAB31 engages FLOTs in lipid raft microdomains to drive EGFR entry into MVEs to form ILVs. Meanwhile, RAB31 recruits TBC1D2B to inactivate RAB7 preventing the fusion of MVEs with lysosomes, thereby enabling that the sequestered EGFR on ILVs are secreted as exosomes (Fig. 9b, c). Therefore, RAB31 occupies the key checkpoint of MVEs for exosome biogenesis and determines the fates of endocytic membrane proteins by balancing with RAB7.

Notably, we propose that the RAB31-FLOTs machinery marks a lipid raft microdomains-dependent exosome pathway that controls RTKs sorting into exosomes. Lipid raft microdomains are fluctuating nanoscale assemblies of sphingolipid, cholesterol and proteins that can be stabilized to coalesce, forming platforms that function in signaling, viral infection and membrane trafficking.^47,48,66–68^ Our findings provide a comprehensive sets of evidence for an ESCRT-independent exosome biogenesis pathway: the scaffold proteins FLOTs coordinate with sphingolipid ceramide and cholesterol to form the platforms; active RAB31 not only as a trigger drives the platforms budding into MVEs to form ILVs, but also recruits TBC1D2B to inactivate RAB7, thereby preventing the fusion of MVEs with lysosomes and enabling the secretion of ILVs as exosomes. The cargoes RTKs can phosphorylate RAB31 to switch RAB31 to be active, thereby driving these RTKs in the platforms entry into MVEs to form ILVs (exosomes). The well-known ESCRT, containing 30 proteins classified into ESCRT-0, I, II and III, as well as auxiliary proteins, are first identified as a large machinery mediating the sorting of ubiquitylated membrane proteins budding into MVEs to form ILVs for lysosomal degradation.^21,23,25,69^ Interestingly, two ESCRT components, Tsg101 and Alix are hijacked by certain proteins, such as Syntenin, containing late domains to mediate their sorting into exosomes.^1,8,10^ Remarkably, we showed that ceramide and cholesterol in lipid raft microdomains are not required for the biogenesis of exosomes driven by the ESCRT machinery. Therefore, we redefine the machineries for the biogenesis of ILVs (exosomes): the simple and universal active RAB31-FLOTs machinery is dependent on lipid raft microdomains, together with the large and circumscribed ESCRT machinery that is independent of lipid raft microdomains. This redefinition is consistent with the two subpopulations of exosomes recently identified;^41^ small exosomes contain concentrated FLOTs and are most likely canonical exosomes, whereas large exosomes contain relatively higher ESCRT components and may represent noncanonical exosomes. Moreover, we further show that RAB31-FLOTs and Syntenin-Alix-ESCRT-III machineries are two parallel exosome pathways, which are responsible for different cargoes. Syntenin-Alix-ESCRT-III pathway is required for syndecan, UNC93B1 and tetraspanins,^10–13^ such as CD9, CD81 and CD63 etc., sorting into exosomes as a basal constitutive secretion, whereas active RAB31-FLOTs machinery that strongly triggered by upstream signals (e.g., EGFR) can drive tetraspanins CD9, CD81 and CD63 as well as their binding partner Syntenin-1 sorting into exosomes bypassing the Alix-ESCRT-III pathway. Because membrane proteins are sorted into lipid raft microdomains to form platforms that function in membrane signaling and trafficking,^47,48^ we speculate that the RAB31-FLOTs machinery may act as a common driver for sorting membrane proteins besides RTKs into exosomes.

Membrane budding is a key step in vesicular transport, MVE biogenesis, and enveloped virus release.^70^ ESCRT and RAB31-FLOTs machineries carry out the budding of MVE membrane to form ILVs, where budding occurs away from the cytosol, therefore, these two different physical machineries mediate reverse-topology membrane budding (recently referred as inverse membrane involution).^69,71^ Such inverse membrane remodeling is mostly mediated by ESCRT, which occurs in a wide range of cellular processes, supporting cytokinesis, endosome maturation, autophagy, membrane repair and many other processes.^69,71^ Similarly, normal-topology or classical membrane budding are also mediated by large coat-protein complexes containing clathrin, coat protein I (COPI) and COPII, as well as mediated by the lipid raft domains, where budding occurs towards the cytosol.^48,70,72–75^ Particularly, RAB31 drives the budding of MVE membrane into the lumen to form ILVs, which is distinct from the membrane budding of vesicle formation mediated by other RAB GTPases.^19^ This work therefore expands on our knowledge of RAB31 in vesicular transport and highlights the important and widespread function of lipid raft microdomains in membrane budding.

In summary, we have shown that RAB31 has dual functions in the biogenesis of exosomes: driving ILVs formation and avoiding MVEs degradation. The conceptual framework provided here is a stepforward in better understanding of the heterogenous biogenesis of exosomes. The defined RAB31-FLOTs and RAB31-TBC1D2B machineries may provide a basis to potentially design therapeutic strategies for human diseases, such as cancer and neurodegenerative diseases.

## Materials and methods

### Plasmids

The cDNA of 62 selected small RAB GTPases were cloned by PCR from the cDNA of HEK-293T cells and was inserted into the pCMV-3× Flag (Sigma) backbone between *EcoR*I and *Sal*I fused with 3× Flag tag at the N-terminus of each RAB. The plasmids containing constitutively active mutations from glutamine (Q) to leucine (L) at the corresponding sites of RAB GTPases were generated by PCR using the forward primers containing mutation sites. These constitutively active cDNAs tagged with Flag were again cloned by PCR and inserted into the pSin-EF2-puro-oligo backbone^76^ between *BstB*I and *Nhe*I. Thus, the constitutively active RAB GTPase library was established within lentiviral overexpression plasmids. The Flag-encoding sequence and wild-type RAB31 (RAB31^WT^) tagged with Flag were also cloned and inserted into pSin-EF2 between *BstB*I and *Nhe*I.

The shRNA expression constructs were in the pLKO.1-puro backbone. The sequences of the shRNAs used in this study were as follows: shRAB31#2 (TRCN0000047733; 5′-CGTGGTTGAGACAAGTGCAAA-3′); shRAB31#4 (TRCN0000379576; 5′-TGCTAAGGAATACGCTGAATC-3′); shHrs#1 (TRCN0000037898; 5′-GCACGTCTTTCCAGAATTCAA-3′); shHrs#3 (TRCN0000037895; 5′-CCTGTACTCTTCACCTGTGAA-3′); shTsg101#1 (TRCN0000007563; 5′-GCCTTATAGAGGTAATACATA-3′); shTsg101#2 (TRCN0000315110; 5′-GCAGAGCTCAATGCCTTGAAA-3′); shAlix#1 (TRCN0000029396; 5′-CCAGAACAAATGCAGTGATAT-3′); shAlix#2 (TRCN0000343595; 5′-CCTGAATTACTGCAACGAAAT-3′); shCD9#1 (TRCN0000057470; 5′-GCTGTTCGGATTTAACTTCAT-3′); shCD9#2 (TRCN0000296958; 5′-CCTGCAATGAAAGGTACTATA-3′); shCD81#1 (TRCN0000300293; 5′-GATCATGATCTTCGAGATGAT-3′); shCD81#2 (TRCN0000300291; 5′-CCTGCTCTTCGTCTTCAATTT-3′); shCD63#1 (TRCN0000007851; 5′-GCAAGGAGAACTATTGTCTTA-3′); shCD63#2 (TRCN0000007850; 5′-GCCTCGTGAAGAGTATCAGAA-3′); shFLOT1 (TRCN0000382424; 5′-GGAAGTACTGGACATTCTAAC-3′); shFLOT2 (TRCN0000280654; 5′-GAAGAGATTGAGATTGAGGTT-3′). The sgSyntenin-1 expression constructs were in the pLentiCRISPR-puro backbone. The sequences of the sgSyntenin-1 used in this study was 5′-AAGTGGTGCACCAGAAACCA-3′.

The cDNAs of CD63, HRS, FLOT1 and FLOT2 were cloned by PCR from the cDNA of HEK-293T cells and were inserted into the pEGFP-N1 (Clontech) backbone between *Hind*III and *Pst*I fused with GFP tag at their C-termini. The fusion cassettes of CD63-, HRS-, FLOT1- and FLOT2-GFP were cloned and inserted into the pCNDA3.1 backbone (Invitrogen) between *Kpn*I and *Xho*I. The cDNA of RAB5A was cloned by PCR from the cDNA of HEK-293T cells and was inserted into the pEGFP-C1 (Clontech) backbone between *Sal*I and *BamH*I fused with GFP tag at the N-terminus of RAB5A. The fusion cassette of EGFR-HA was cloned by PCR from the cDNA of HeLa cells with an HA-encoding sequence in the reverse primer and was inserted into pCNDA3.1 between *Kpn*I and *Xho*I. The pCNDA3.1-HA plasmids containing EGFR mutations L858R (M1), L858R/T790M (M2), L858R/T790M/C797S (M3), Del E746-A750 (D1), Del E746-A750/T790M (D2) and Del E746-A750/T790M/C797S (D3) were generated by PCR using the forward primers containing mutation sites step by step. The cDNAs of HER2, IGF1R, PDGFR-α, PDGFR-β, FGFR-1, FGFR-2, MET and NTRK2 were cloned from the cDNA of HEK-293T, HeLa or HGC27 cells and were inserted into pCNDA3.1-HA between *Kpn*I and *EcoR*I. The pCMV-3Flag plasmids containing RAB31 tyrosine mutations Y75F, Y76F, Y86F, Y95F, Y137F, Y76/86F, Y76/137F, Y86/137F and Y76/86/137F (3YF) were generated by PCR using the forward primers containing mutation sites step by step. The pCMV-3Flag plasmids containing somatic mutations of RAB31 in human cancer were generated by PCR using the forward primers containing mutation sites. The pCMV-3Flag plasmids containing shRNA-resistant cDNAs of RAB31 were generated by PCR using the forward primers containing three nonsense mutation sites (5′-C GTG GTT GAG AC**G** AG**C** GC**G** AA-3′) targeted by shRAB31#2. The mutated cassettes of the indicated EGFR-HA and Flag-RAB31 were cloned and inserted into pSin-EF2 between *BstB*I and *Nhe*I to generate lentiviral overexpression plasmids.

The fusion cassette FLOT1-HA was cloned by PCR from the cDNA of HEK-293T cells with the *EcoR*I site following the HA-encoding sequence in the reverse primer and was inserted into pCNDA3.1 between *Kpn*I and *Xho*I. The cDNAs of FLOT2, erlin1, erlin2, prohibitin1, prohibitin2, stomatin and STOML3 were cloned from the cDNA of HEK-293T cells and were inserted into pCNDA3.1-HA between *Kpn*I and *EcoR*I. The fusion cassette of FLOT1-Flag was cloned by PCR with *EcoR*I site following the Flag-encoding sequence in the reverse primer and was inserted into pCNDA3.1 between *Kpn*I and *Xho*I. The cDNA of FLOT2 was cloned and inserted into pCNDA3.1-Flag between *Kpn*I and *EcoR*I. The indicated truncations and mutations of FLOT1 (aa 1–362, SPFH domain (1–189), flotillin1 domain (190–427), Del aa 71–100, Del AH1 (aa 78–91), AH1-M1 (^78^KEML^81^ mutated to AAAA) and AH1-M2 (^88^FLGK^91^ mutated to AAAA)) and FLOT2 (aa 1–365, SPFH domain (1–192), flotillin2 domain (192–428), Del aa 71–100, Del AH1 (aa 81–94), Del AH2 (aa 94–109), AH2-M4 (both ^96^VQDI^99^ and ^104^LQTL^107^ mutated to 8 A)) were generated by PCR using the forward primers containing mutation sites. The chimeric cassettes of sto-flotillin1 (1–228 aa of stomatin fused with aa 190–427 of FLOT1) and sto-flotillin2 (1–228 aa of stomatin fused with aa 193–428 of FLOT2) were cloned and inserted into pCNDA3.1-HA between *Kpn*I and *EcoR*I. The plasmids containing shRNA-resistant cDNAs of FLOT1, FLOT1-AH1M1, and sto-flotillin1 were generated by PCR using the forward primers containing three nonsense mutation sites (5′-G GAA GTA CTG GA**T** AT**C** CT**C** AC-3′) targeted by shFLOT1. The plasmids containing shRNA-resistant cDNAs of FLOT2, FLOT2-AH2M4, and sto-flotillin2 were generated by PCR using the forward primers containing three nonsense mutation sites (5′-GAA GAG ATT GAG AT**C** GA**A** GT**G**-3′) targeted by shFLOT2. The mutated cassettes and indicated shRNA-resistant cDNAs were cloned and inserted into pSin-EF2 between *BstB*I and *Nhe*I to generate lentiviral overexpression plasmids. The cDNA of GFP was also cloned and inserted into pSin-EF2. The cDNA of RAB31 and its mutants was also cloned and inserted into pSin-EF2-GFP to generate pSin-EF2-GFP-RAB31 variants. The V5-coding sequence, TBC1D2A, TBC1D2B, TBC1D5 and TBC1D15 were cloned and inserted into pSin-EF2 between *BstB*I and *Nhe*I to generate pSin-EF2-V5-TBC1 family plasmids. The pSin-EF2-V5-TBC1D2B plasmid containing various truncations of TBC1D2B were generated by PCR. The streptavidin-binding peptide (SBP)-encoding sequence and RILP were cloned and inserted into pCNDA3.1 between *Kpn*I and *Xho*I to generate pCNDA3.1-SBP-RILP. The HA-encoding sequence and RILP were cloned and inserted into pSin-EF2 between *BstB*I and *Nhe*I to generate pSin-EF2-HA-RILP plasmids.

All the above-described constructs were fully verified by sequencing.

### Antibodies and reagents

The following antibodies were used for western blotting: Flag rabbit antibody (1:2000; Sigma; F7425), Flag rabbit antibody (1:2000; Cell Signaling; 14793), Flag mouse antibody (1:2000; Cell Signaling; 8146), HSP70 mouse antibody (1:5000; Santa Cruz; sc-24;), ubiquitin rabbit antibody (1:1000; Cell Signaling; 3933), HA rabbit antibody (1:2000; Cell Signaling; 3724), HA mouse antibody (1:2000; Cell Signaling; 2367), V5 rabbit antibody (1:2000; Cell Signaling; 13202), GAPDH rabbit antibody (1:2000; Proteintech; 10494–1-AP), EGFR rabbit antibody (1:1000; Cell Signaling; 4267), p-Y845 EGFR rabbit antibody (1:1000; Cell Signaling; 6963), p-Y1068 EGFR rabbit antibody (1:2000; Cell Signaling; 3777), p-Y1173 EGFR rabbit antibody (1:1000; Cell Signaling; 4407), MET rabbit antibody (1:1000; Cell Signaling; 8198), HER2 rabbit antibody (1:1000; Cell Signaling; 4290), Phosphotyrosine rabbit antibody (p-Tyr) (1:1000; Sigma; T1325), Phosphotyrosine rabbit antibody (p-Tyr-1000) (1:2000; Cell Signaling; 8954), p-S473 AKT rabbit antibody (1:1000; Cell Signaling; 4060), AKT rabbit antibody (1:1000; Proteintech; 10176-2-AP), p-ERK1/2 rabbit antibody (1:2000; Cell Signaling; 4370), ERK2 rabbit antibody (1:1000; Proteintech; 16447-1-AP), RAB31 rabbit antibody (1:500; Proteintech; 16182-1-AP), RAB31 rabbit antibody (1:2000; Sigma; HPA019717), TBC1D2B mouse antibody (1:2000; Santa Cruz; sc-398906), RAB7A rabbit antibody (1:1000; Proteintech; 55469-1-AP), FLOT1 rabbit antibody (1:1000; Cell Signaling; 18634), FLOT2 rabbit antibody (1:1000; Cell Signaling; 3436), CD9 rabbit antibody (1:1000; Cell Signaling; 13403), CD9 mouse antibody (1:200; Santa Cruz; sc-13118), CD81 mouse antibody (1:200; Santa Cruz; sc-166029), CD63 rabbit antibody (1:1000; Abcam; ab134045), CD63 mouse antibody (1:200; Santa Cruz; sc-5275), Hrs rabbit antibody (1:1000; Proteintech; 10390-1-AP), Tsg101 rabbit antibody (1:1000; Sigma; HPA006161), Alix rabbit antibody (1:1000; Proteintech; 12422-1-AP), Syntenin-1 rabbit antibody (1:1000; Proteintech; 22399-1-AP), VPS4 rabbit antibody (1:2000; Proteintech; 17673-1-AP), HSP90 mouse antibody (1:1000; Santa Cruz; sc-13119), β-tubulin rabbit antibody (1:1000; Cell Signaling; 2128), β-actin rabbit antibody (1:1000; Cell Signaling; 4970), Histone H3 rabbit antibody (1:2000; Cell Signaling; 4499).

Antibodies were used for immunoprecipitation: EGFR rabbit antibody (1:100; Cell Signaling; 4267), RAB31 rabbit antibody (1:100; Sigma; HPA019717), FLOT1 rabbit antibody (1:100; Cell Signaling; 18634), FLOT2 rabbit antibody (1:100; Cell Signaling; 3436), TBC1D2B mouse antibody (1:50; Santa Cruz; sc-398906), protein A agarose (Sigma; P3476), protein A/G agarose (Santa Cruz; sc-2003), normal rabbit IgG (1:100; Proteintech; 30000-0-AP), normal mouse IgG (1:200; Santa Cruz; sc-2025), mouse monoclonal Anti-Flag M2 Affinity Gel (Sigma; A2220), mouse monoclonal Anti-HA antibody Agarose (Sigma; A2095), and mouse monoclonal anti-phosphotyrosine antibody agarose (Sigma; A1806). Secondary antibodies were used for immunoblotting: both anti-rabbit horseradish-peroxidase (HRP)-conjugated antibody (Promega; W401B) and anti-mouse HRP-conjugated antibody (Promega; W402B) were diluted at 1:50,000.

The following primary antibodies were used for immunofluorescence: Flag rabbit antibody (1:500; Cell Signaling; 14793), Flag mouse antibody (1:500; Cell Signaling; 8146), HA rabbit antibody (1:500; Cell Signaling; 3724), HA mouse antibody (1:500; Cell Signaling; 2367), V5 rabbit antibody (1:500; Cell Signaling; 13202), EGFR rabbit antibody (1:100; Cell Signaling; 4267), RAB31 rabbit antibody (1:50; GeneTex; GTX55929), TBC1D2B mouse antibody (1:50; Santa Cruz; sc-398906), RAB7 rabbit antibody (1:200; Abcam; ab137029), CD63 mouse antibody (1:500; Santa Cruz; sc-5275), EEA1 rabbit antibody (1:300; Cell Signaling; 3288), LAMP1 rabbit antibody (1:300; Cell Signaling; 9091), LAMP1 mouse antibody (1:50; Santa Cruz; sc-20011). The following secondary antibodies for immunodetection were purchased from Invitrogen and diluted at 1:500: Goat anti-rabbit Alexa Fluor-488 (Molecular Probes; A11034), Goat anti-mouse Alexa Fluor-594 (Molecular Probes; A11032), Goat anti-mouse Alexa Fluor-488 (Molecular Probes; A32723), Goat anti-rabbit Alexa Fluor-594 (Molecular Probes; A11037), Goat anti-mouse Alexa Fluor-647 (Molecular Probes; A21236), Goat anti-rabbit Alexa Fluor-568 (Molecular Probes; A11036).

The following growth factors were used: Human EGF (PeproTech; 96-AF-100-15-500), Human bFGF (PeproTech; 96-100-18B-10), Human IGF-I (PeproTech; 96-100-11-100), Human PDGF-AB (PeproTech; 96-100-00AB-2), Human HGF (PeproTech; 96-100-39H-5). All factors were suspended in sterile-filtered water at the appropriate concentration in stock. The following EGFR-TKIs were used: afatinib (Selleck; S1011), WZ4002 (Selleck; S1173), Erlotinib HCl (OSI-744) (Selleck; S1023), lapatinib (Selleck; S2111), and PD153035 HCl (Selleck; S1079). All EGFR-TKIs were suspended in dimethyl sulfoxide (DMSO) (Sigma; D2650) at the concentration of 10 mM in stock. GW4869 (Sigma; D1692) was suspended in DMSO at the concentration of 350 μM in stock. Simvastatin (Selleck; S1792) and Lovastatin (Selleck; S2061) were suspended in DMSO at the concentration of 10 mM in stock. Flag peptide (ApexBio; A6002) and HA peptide (ApexBio; A6004) were suspended in sterile-filtered water at the concentration of 5 mg/mL in stock. Additionally, 10× kinase buffer (9802) and 10 mM ATP (9804) were purchased from Cell Signaling.

### Cell lines

All cell lines used in this study were originally obtained from the ATCC as follows: HEK-293T, HeLa, NCI-H1975, PC9, A549, NCI-H460, NCI-H1993, MDA-MB231, HCT116, HGC27, EC109, BxPC3, A431, U138, U2OS, A375, PC3 and Hep3B. The cell lines were cultured in DMEM (Gibco) with 10% fetal bovine serum (FBS), 100 U/mL of penicillin and 100 μg/mL of streptomycin and were maintained in a humidified, 5% CO_2_ atmosphere at 37 °C. Cell lines were tested for mycoplasma contamination and were authenticated by the STR method.

### Lentivirus and stable cell line construction

Lentiviral production for shRNA, sgRNA expression or overexpression was performed as follows. HEK-293T cells were seeded into one plate of six-well plates. The following day, cells in each plate were transfected with 3 μg of pLKO.1-shRNA or 3–6 μg of pSin-EF2-cDNA, 2 μg of psPAX2 (*gag*, *pol*) and 1 μg of pMD2G using 24 μL of polyethylenimine (PEI) (2 mg/mL). Viral supernatants were collected 48 h after transfection and were filtered through 0.45-μm PVDF filters (Millipore). For one level of lentiviral transduction, cells were infected with appropriate viruses in six-well plates in the presence of 10 μg/mL polybrene (Sigma) and centrifuged at 2000 rpm (800× *g*) for 60 min (NCI-H1975 cells) or 100 min (HeLa and other cell lines) at 37 °C. After 24 h, medium containing puromycin (0.5 μg/mL) was added, and cells were selected for 72 h. The levels of endogenous and overexpressed proteins were then verified by western blotting.

For two levels of lentiviral transduction, the indicated gene-knockdown HeLa cells were second infected with viruses of overexpression of RAB31^Q65L^ to generate gene-knockdown plus RAB31^Q65L^-overexpressed cells. The double FLOT1- and FLOT2-knockdown plus RAB31^Q65L^-overexpressed HeLa cells were infected with the indicated viruses of the overexpression of various shRNA-resistant FLOT to generate FLOT-rescued cells. The NCI-H1975 cells were infected with the indicated viruses of the overexpression of shRNA-resistant RAB31 and then second infected with the virus of shRAB31#2 to generate RAB31-rescued cells. The levels of endogenous and overexpressed proteins were then verified by western blotting.

### Serum starvation and ligand re-stimulation

HEK-293T or HeLa cells were seeded into appropriate plates 1 day before experiments. On the next day, cells were transfected or were untransfected with the indicated plasmids. After 24 h, the cells were rinsed twice with PBS; for serum starvation, cells were incubated with DMEM without FBS for 24 or 48 h depending on the objective. For ligand re-stimulation, serum-starved cells were re-stimulated with DMEM containing appropriate ligands at the indicated times. After the treatment, cells were prepared for western blotting, immunoprecipitation or immunofluorescence, and the cultured supernatants were collected for exosome isolation.

### Inhibition assays

For the inhibition of EGFR kinase activity, serum-starved HEK-293T cells were treated with DMEM containing 1 μM EGFR-TKIs for 4 h. After treatment, the cells were prepared for western blotting and immunoprecipitation. HeLa cells were treated with DMEM containing 1 μM EGFR-TKIs for 24 h. After the treatment, the cells were prepared for western blotting, immunoprecipitation or immunofluorescence, and the cultured supernatants were collected for exosome isolation. For the inhibition of nSMase or 3-hydroxy-3-methyl glutaryl coenzyme A reductase activity, HeLa cells were treated with DMEM containing 5 μM GW4869, 5 μM simvastatin or 10 μM lovastatin for 20 h.^14^ After treatment, the cells were prepared for immunofluorescence, and the cultured supernatants were collected for exosome isolation.

### Western blotting and immunoprecipitation

For western blotting, the cells were washed once in cold PBS and then were lysed on ice in RIPA buffer (50 mM Tris-HCl, pH 7.5, 150 mM NaCl, 1 mM EDTA, 1% NP40) containing Protease Inhibitors Cocktails set I (Calbiochem; 539131) and Phosphatase Inhibitor Cocktails set II (Calbiochem; 524625). The lysates were cleared by centrifugation at 14,000× *g* for 10 min at 4 °C. For immunoprecipitation (IP) or phospho-tyrosine IP, the anti-Flag, anti-HA beads or anti-phospho-tyrosine beads (Sigma) were washed three times with RIPA buffer. Subsequently, 20 μL of the beads was added and incubated with the lysates overnight at 4 °C. For endogenous IP, protein A or protein A/G agarose beads were washed three times with RIPA buffer, and then EGFR, RAB31, FLOT1, FLOT2 and TBC1D2B antibody or the control rabbit or mouse IgG were added into the NCI-H1975 cell lysates with the washed agarose, followed by incubation overnight at 4 °C. The beads were washed five times with RIPA buffer. The IPs and cell lysates were then boiled in gel loading buffer for 10 min and resolved by 10% or 12% SDS-PAGE depending on the molecular mass of the target proteins. The gels were transferred to Immobilon-P PVDF membranes (Millipore), which were then blocked in PBS with 5% nonfat milk and 0.1% Tween-20 and probed with primary antibodies overnight at 4 °C. Secondary HRP-conjugated antibodies were used, and clarity ECL substrate (Bio-Rad) or high-sig ECL substrate (Tanon) was used for detection by MiniChmei Chemiluminescence imager (SAGECREATION, Beijing).

### Streptavidin pull-down assay

HEK-293T cells were transfected with the plasmids expressing SBP-RILP and Flag-RAB31 with or without EGFR^M2^-HA or V5-TBC1D2B. After 36 h, the cells were washed once in cold PBS and then were lysed on ice in RIPA buffer containing protease and phosphatase inhibitors. The lysates were cleared by centrifugation at 14,000× *g* for 10 min at 4 °C. Streptavidin sepharose beads (GE Healthcare, 17-5113-01) were washed three times with RIPA buffer. Subsequently, 20 μL of the beads was added and incubated with the lysates for 1.5 h at 4 °C. The beads were washed five times with RIPA buffer. The pull-down (PD) of active RAB7, SBP-RILP and whole cell lysates were detected by western blotting and gel staining with Coomassie brilliant blue (CBB) R250.

### GTP-binding assay

For binding of RAB31 and RAB7 to GTP-Agarose beads, the RAB31 knockdown or ectopic expression cells were harvested on ~90% confluency. Cells were suspended in binding buffer (20 mM HEPES pH 8, 150 nM NaCl, 10 mM MgCl_2_) containing a cocktail of protease and phosphatase inhibitors and lysed using three freeze thaw cycles, then centrifuged at 14,000× *g* and the supernatants were incubated with 100 μL of GTP-Agarose suspension (Sigma Aldrich, G9768) for 1.5 h with rotation at 4 °C. The beads were pelleted by centrifugation, washed three times in binding buffer and suspended in 40 μL SDS-PAGE sample buffer. The proteins were boiled and subjected to SDS-PAGE and western blotting.

### Immunofluorescence

Cells were seeded into glass-bottomed culture dishes (NEST Biotechnology; 801002) 1 day before experiments. All transfection experiments were performed using Lipofectamine 2000 or Lipofectamine 3000 (Invitrogen). The HeLa cells that overexpressed selected constitutively active RAB GTPase were transfected with EGFR-HA for 24 h, and various cancer cell lines overexpressing RAB31^Q65L^ were transfected with EGFR-HA for 24 h, and then the cells were treated under serum starvation for 24 h. The indicated various stable HeLa cells were transfected with EGFR-HA and CD63-GFP for 24 h, and then the cells were treated under serum starvation for 24 h. The indicated various stable NCI-H1975 cells were treated under serum starvation for 24 h, and then endogenous EGFR or CD63 with overexpressed Flag-RAB31 was detected. At the end of various treatments, the cells were rinsed twice with PBS and were fixed for 15 min with 4% paraformaldehyde in PBS at room temperature. The cells were rinsed twice with PBS and permeabilized with 0.5% Triton X-100 in PBS for 15 min. After rinsed twice with PBS, the cells were incubated with goat serum for 30 min at room temperature. Next, the cells were incubated with the primary antibodies for 2 h at room temperature or overnight at 4 °C. After rinsed three times (15 min every time) with PBS, the cells were incubated for 2 h at room temperature with the following secondary antibodies: anti-mouse Alexa Fluor-594, anti-rabbit Alexa Fluor-488, anti-mouse Alexa Fluor-488, anti-rabbit Alexa Fluor-594, anti-mouse Alexa Fluor-647, or anti-rabbit Alexa Fluor-568, (Molecular Probes, Invitrogen). Nuclei were stained with Hoechst 33342 for 2 min (Molecular Probes, Invitrogen). After rinsing three times (15 min every time) with PBS, the cells were mounted using antifade mounting medium (Invitrogen). The cells were imaged using laser scanning confocal microscopes (Olympus, IX83, FV1000, 60× oil lens; ZEISS, LSM880, ZEN2.6, 63× oil lens).

The ratio of co-localization is quantified by this basic rule: the number of A vesicles co-localized with B vesicles divided by the total number of B vesicles in one field, e.g., the number of EGFR-HA vesicles co-localized with CD63-positive LE/MVEs divided by the total number of CD63-positive LE/MVEs in one field, which is the ratio of co-localization of EGFR-HA with CD63-positive LE/MVEs. Similarly, the number of CD63-GFP-positive MVEs containing EGFR-HA, FLOT-HA or Flag-RAB31^Q65L^ divided by the total number of CD63-GFP-positive MVEs in one field, which is the ratio of entry of EGFR-HA, FLOT-HA or Flag-RAB31^Q65L^ into CD63-GFP-positive MVEs. The diameter of CD63-positive LE/MVEs is quantified by equivalent scale conversion in the enlarged image.

### Structured illumination microscopy

Structured illumination microscopy (SIM) super-resolution images were taken using a Nikon N-SIM system with a 100× oil immersion objective lens, 1.49 NA (Nikon). Images were captured using Nikon NIS-Elements and were reconstructed using slice reconstruction in NIS-elements. Images of fixed cells for SIM were taken at a single *Z*-plane, and images of fixed cells for 3D-SIM were taken using *Z*-stacks with step sizes of 0.12 μm.

### IEM

Cells were prepared for IEM with LR White resin (14381-UC, ELECTRON MICROSCOPY SCIENCES) as previously described,^77^ with some modifications. Briefly, cells were pelleted at 150× *g* for 8 min and fixed in a solution containing 2% paraformaldehyde, 0.05% glutaraldehyde and 0.1 M PBS (pH 7.4) for 90 min at 4 °C. The fixed pellets were washed three times with 0.1 M PBS (pH 7.4) for 10 min at 4 °C and then dehydrated at –20 °C with a 30%, 50%, 70%, and 90% graded ethanol series; each ethanol step lasted for 20 min, and the 30% ethanol dehydration step occurred at 4 °C. Samples were infiltrated with 40%, 70%, and 100% LR White-ethanol series at −20 °C for 1 h per step, followed by infiltration with 100% LR White at –20 °C overnight. The resin containing sample was then polymerized in PCR tube by UV irradiation (360 nm) at –20 °C for 72 h and at room temperature for 48 h. Immunolabeling was performed with a rabbit anti-HA antibody (1:20; Cell Signaling; 3724) or mouse anti-Flag antibody (1:20; Cell Signaling; 8146) for 2 h at 37 °C, followed by incubation with goat anti-rabbit IgG conjugated to 10-nm gold particles (1:20; Sigma; G7402) or goat anti-mouse IgG conjugated to 10-nm gold particles (1:20; Sigma; G7777) as the secondary antibody for 2 h at 37 °C. The samples were visualized with a JEOL JEM-1400 transmission electron microscope at the accelerating voltage of 120 kV with the AMT XR41 digital imaging system.

### Concentrated conditional media, characterization and analyses

Concentrated conditional media mainly containing EVs were obtained by ultrafiltration as previously described.^40,78,79^ Briefly, cells were seeded into 15-cm plates in DMEM with 10% FBS until they reached a confluency of 80%–90%. The cells were rinsed twice with PBS and were cultured in 30 mL of DMEM without FBS for 24 or 48 h. The cultured supernatants were collected and subjected to sequential centrifugation steps (600× *g* for 10 min; 2000× *g* for 30 min) to discard cells and cellular debris at 4 °C. Next, the supernatants were filtered through 0.22-μm PVDF filters (Millipore). The supernatants mainly containing small EVs and other extracellular matter were further concentrated to 200 μL by 100 K NMWL centrifugal filtration (Amicon Ultra-15; Millipore), and then 15 mL of PBS was added into the Amicon and further concentrated to 150~200 μL twice. Generally, for each stable cell line, 60 mL of media from two 15-cm plates were used to obtain the concentrated conditional media. The concentrated conditional media were lysed with equal volumes of 2× RIPA buffer, and the protein concentration of lysates were quantified by BCA assay. 10 μg of total protein in lysates were subjected to SDS-PAGE and western blotting. EGFR, RAB31 and the well-known EV markers (FLOT1, FLOT2, CD9, CD81, CD63, Tsg101, Alix, GAPDH and HSP70) were detected to determine and analyze the EV components in the concentrated conditional media. The EV size and particle number were analyzed using the NS300 nanoparticle characterization system (NanoSight, Malvern Instruments) equipped with NTA 3.2 analytical software. For electron microscopy using negative staining, the concentrated conditional media were dropped onto formvar stabilized with carbon support film-coated copper grids for 2 min, and then the grids were allowed to dry and stained for contrast using lead citrate for 1 min. The samples were imaged on a JEM1400 (JEOL) at 120 kV with an AMT XR41 digital imaging system.

### High-resolution (12%–36%) iodixanol density gradient fractionation

The 600 mL cultured supernatants of NCI-H1975 or MDA-MB231 cells were performed ultrafiltration to obtain the concentrated conditional media. The concentrated conditional media were separated by iodixanol density gradient fractionation to isolate small EVs with NV components as previously described.^6^ Briefly, iodixanol (OptiPrep) density media (Sigma Aldrich, D1556) were prepared in ice-cold PBS immediately before use to generate discontinuous step (12%–36%) gradients. The concentrated conditional media were resuspended in ice-cold PBS and mixed with ice-cold iodixanol/PBS for a final 36% iodixanol solution. The suspension was added to the bottom of a centrifugation tube and solutions of descending concentrations of iodixanol in PBS were carefully layered on top yielding the complete gradient. The bottom-loaded 12%–36% gradients were subjected to ultracentrifugation at 120,000× *g* for 15 h at 4 °C using a SW41 TI rotor (Beckman Coulter). Twelve individual fractions of 1 mL were collected from the top of the gradient. Each individual 200 μL fraction was transferred to new tubes, then added 50 μL 5× loading buffer and boiled for 10 min, each individual 20 μL sample was subjected to SDS-PAGE and western blotting. RAB31, FLOT1, FLOT2, EGFR, CD9, CD81, CD63, Syntenin-1, Tsg101, Alix, VPS4, GAPDH, HSP70, HSP90, β-tubulin, β-actin, Histone H3 were detected to determine and analyze the small EVs and NV components.

### Protein purification and in vitro kinase assay

HEK-293T cells were individually transfected with Flag-RAB31 and EGFR-HA. After 36 h, the cells were serum starved for 4 h and lysed with RIPA buffer, and then cell lysates of two wells of six-well plates were combined into one tube. The anti-Flag and anti-HA beads were used to immunoprecipitate the proteins. The washed beads were resuspended with 250 μL of 1× kinase buffer containing Flag or HA peptides (200 μg/mL) and were rotated for 6 h at 4 °C to elute the proteins. Each 50 μL of eluted RAB31 or EGFR was added into a new tube in the presence of 200 μM ATP. The kinase reaction was incubated at 30 °C for 30 min. At the end of the reaction, the samples were boiled in gel loading buffer for 10 min and were resolved by SDS-PAGE. Tyrosine phosphorylation of RAB31 was detected using anti-phospho-tyrosine antibody (p-Tyr-1000) by immunoblotting analysis. The protein purity was identified via gel staining with CBB R250.

### Mass spectrometry analysis

After in vitro kinase reaction, the prepared protein samples were separated by SDS-PAGE and stained with CBB R250. The bands of RAB31 were excised and sent to Wininnovate Biotechnology Co. Ltd. in Shenzhen for protein phosphorylation identification. The peptides were analyzed by liquid chromatography–tandem mass spectrometry on a Triple TOF 6600 tandem mass spectrometer (Sciex, Concord, Ontario, Canada).

### Co-culture assay

NCI-H1975 cells and PC9-GFP cells were simultaneously seeded into six-well plates (5000 per well of every cell line) and co-cultured for 10 days. The medium was replaced with DMEM containing 1 μM erlotinib and was further cocultured for 5 days. The dead cells and cellular debris were removed. After rinsed twice with PBS, the cells were cultured in DMEM. The GFP-positive clones were imaged on an inverted fluorescence microscope (Olympus, IX73) with the 4× lens. The number of cells in 20 clones of each group were counted. Unpaired *t*-test was used to test differences between the group of WT, Q65L, R77Q or 3YF with group Vector.

### The concentrated conditional media treatment

PC9-GFP cells were seeded into 12-well plates (3000 per well). At 1, 3, 5, 7th day, the old medium was replaced with fresh medium containing PBS or 10 μg of the concentrated conditional media that were purified from NCI-H1975 stable cell lines. At 9th day, the medium was replaced with DMEM containing 1 μM erlotinib and was further cocultured for 6 days. The dead cells and cellular debris were removed. After rinsing twice with PBS, the cells were cultured in DMEM. The GFP-positive clones were imaged on an inverted fluorescence microscope (Olympus, IX73) with the 10× lens. The number of cells in indicated number of clones in each group were counted. Unpaired *t*-test was used to test differences between the two groups.

### The small EV treatment

First, each 450 mL cultured supernatants of NCI-H1975 Vector, RAB31^WT^ and RAB31^3YF^ stable cells were performed ultrafiltration to obtain the concentrated conditional media. The concentrated conditional media were separated by iodixanol density gradient fractionation to isolate small EVs with NV material. The top six fractions containing small EVs were collected, mixed into 100 mL PBS and further concentrated by ultrafiltration to obtain pure small EVs. PC9-GFP cells were seeded into 12-well plates (3000 per well). At 1, 3, 5, 7th day, the old medium was replaced with fresh medium containing 5 μg of the pure small EVs that were purified from NCI-H1975 stable cell lines. At 9th day, the medium was replaced with DMEM containing 1 μM erlotinib and was further cocultured for 6 days. The dead cells and cellular debris were removed. After rinsing twice with PBS, the cells were cultured in DMEM. The GFP-positive clones were imaged on an inverted fluorescence microscope (Olympus, IX73) with the 10× lens. The number of cells in indicated number of clones in each group were counted. Unpaired *t*-test was used to test differences between the two groups.

### Statistical analysis, graphing and figure assembly

Quantification analyses of protein expression were analyzed using ImageJ software. Statistical analyses of the data were analyzed using Prism 8 (GraphPad) software. Differences between two groups were assessed by unpaired two-sample *t*-test. The secondary structures of RAB31, FLOT1 and FLOT2 were predicted by the Phyre2 web portal.^80^ The domain structures of TBC1D2B were illustrated by GPS-DOG 2.0.^81^ All final figures were assembled in Illustrator (Adobe).

## Supplementary information

Supplementary information, Fig. S1

Supplementary information, Fig. S2

Supplementary information, Fig. S3

Supplementary information, Fig. S4

Supplementary information, Fig. S5

Supplementary information, Fig. S6

Supplementary information, Fig. S7

Supplementary information, Fig. S8

Supplementary information, Fig. S9

Supplementary information, Fig. S10

Supplementary information, Fig. S11
