# Supplementary material for: RAB31 marks and controls an ESCRT-independent exosome pathway
Source: Cell Res. 2020 Sep 21;31(2):157–77. doi: 10.1038/s41422-020-00409-1 (PMC8027411; doi:10.1038/s41422-020-00409-1)
Supplement: Supplementary file 1 — Supplementary information, Fig. S1 [file 41422_2020_409_MOESM1_ESM.pdf]

Supplementary information, Fig. S1

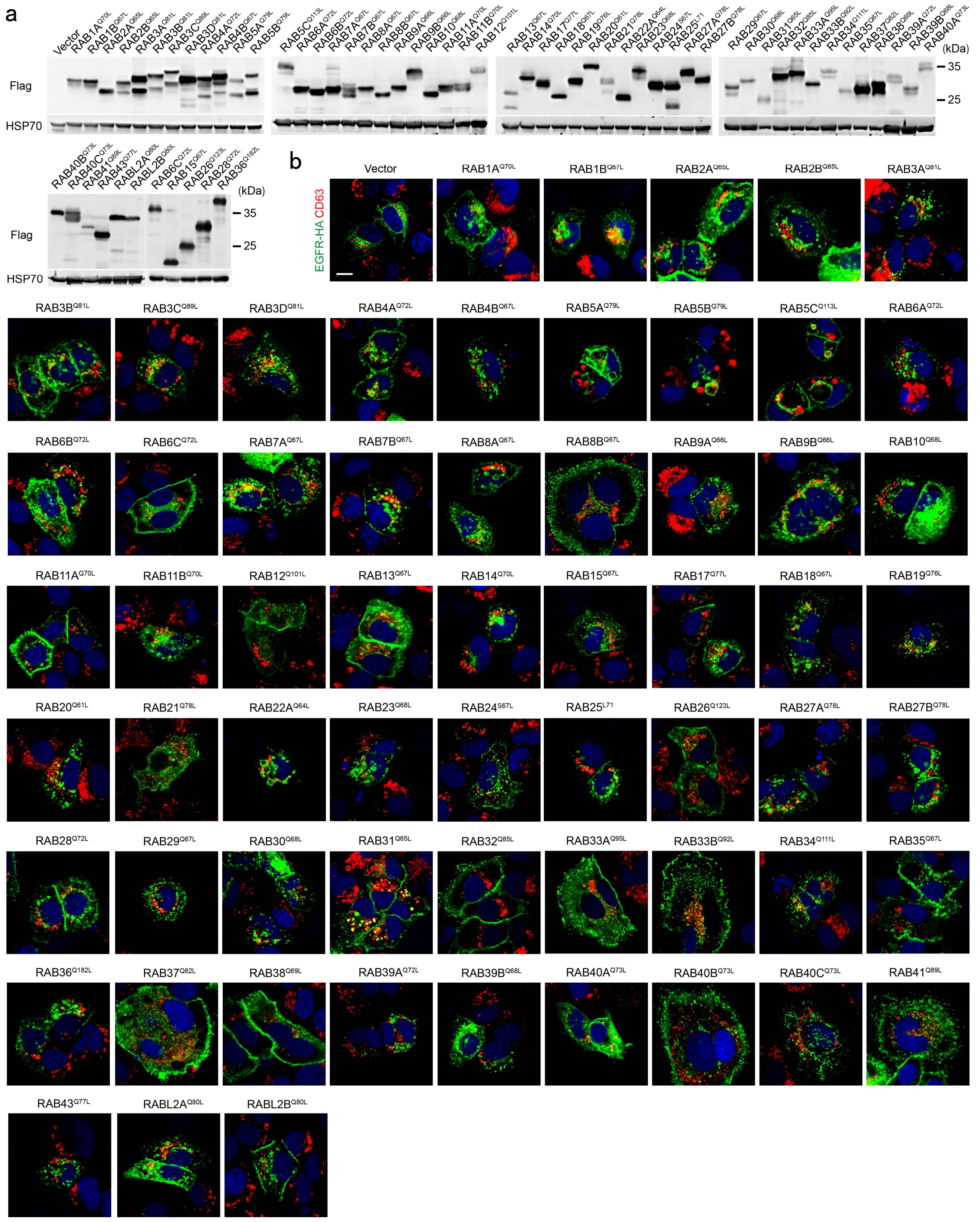

Supplementary information, Fig. S1. RAB31^{Q65L} specifically directs EGFR localization to CD63-positive MVEs. a Western blotting analyses of whole-cell lysates from HeLa cells stably expressing the indicated constitutively active RAB GTPase. **b** Immunofluorescence of EGFR-HA (green) with CD63 (red) in the cells used in (**a**) transiently expressing EGFR-HA under normal condition. Scale bar, 10 μm.
