# Supplementary material for: RAB31 marks and controls an ESCRT-independent exosome pathway
Source: Cell Res. 2020 Sep 21;31(2):157–77. doi: 10.1038/s41422-020-00409-1 (PMC8027411; doi:10.1038/s41422-020-00409-1)
Supplement: Supplementary file 2 — Supplementary information, Fig. S2 [file 41422_2020_409_MOESM2_ESM.pdf]

Supplementary information, Fig. S2

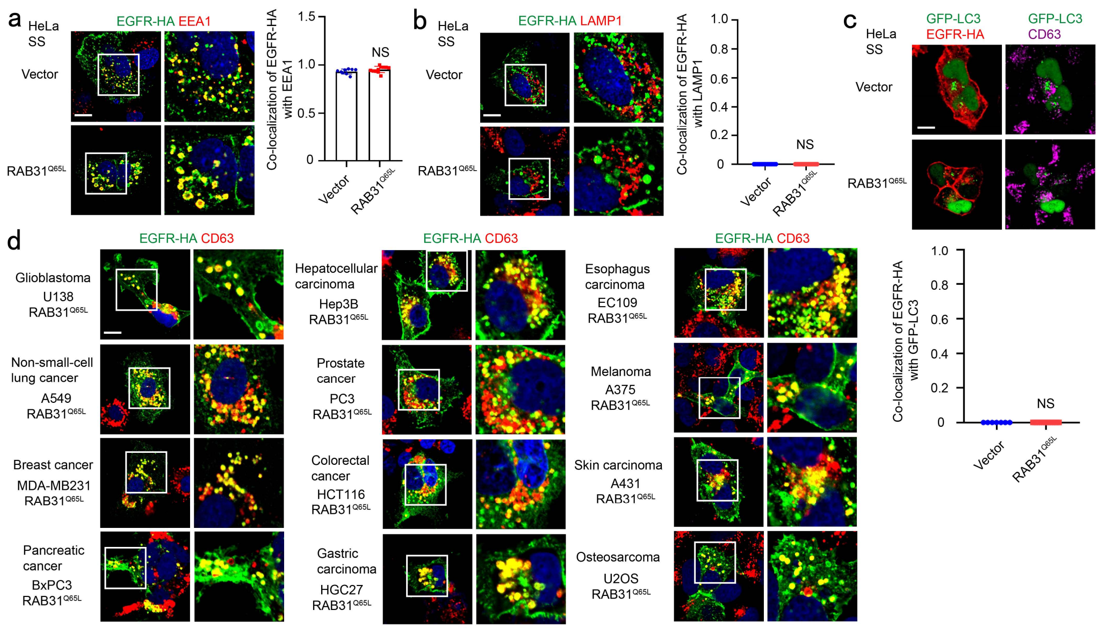

e Somatic mutations of RAB31 in human cancers

Type of cancers	Mutation sites
Constitutively active	Q65L
Skin cutaneous melanoma	G13W, Q28H, R67L, L58F, W60R, Q91H, P169L, G179E
Cervical squamous cell carcinoma and endocervical adenocarcinoma	R25Q, S42F, K135N, K98N, K102E, V145M, E157K, L159I, I167F, N175H, N177S
Lung adenocarcinoma and small cell lung cancer	P37H, R77Q, S20L, A72D
Hepatocellular carcinoma	G64V, R165S, G40E, A117D
Breast invasive cancer	A41P, H30D, P130T
Prostate cancer	A138T, A82V
Colorectal adenocarcinoma	F160C, F43V, K90T, G118V, R165C
Skin cutaneous carcinoma	E66Q, H69Y, G118E, P169H, P169S
Esophagus carcinoma	V23M, T45A
Renal carcinoma	M114I

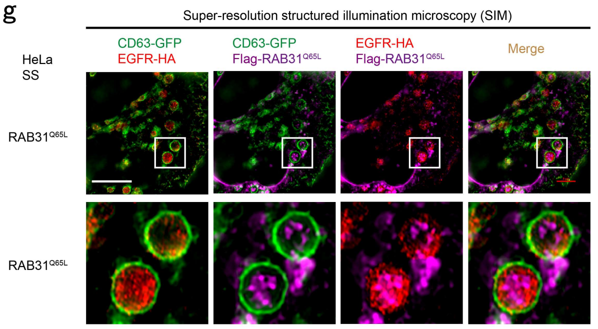

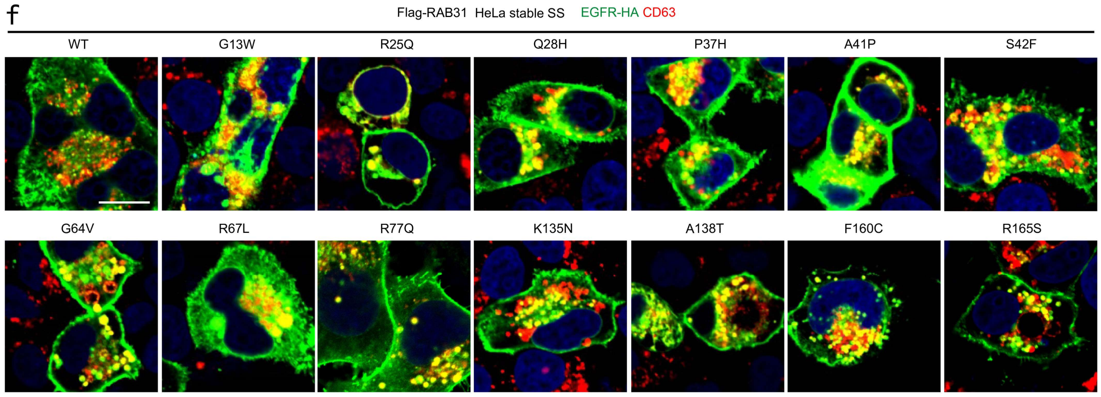

Supplementary information, Fig. S2. Various active RAB31 generally directs EGFR localization to CD63-positive MVEs. **a** Left, immunofluorescence of EGFR-HA (green) with EEA1 (red) in the indicated stable HeLa cells transiently expressing EGFR-HA under serum starvation (SS). Right, the ratio of co-localization of EGFR-HA with EEA1-positive vesicle in Vector (*n* = 9 fields) and RAB31^{Q65L} (*n* = 11 fields). **b** Left, immunofluorescence of EGFR-HA (green) with LAMP1 (red) in the indicated stable HeLa cells transiently expressing EGFR-HA under SS. Right, the ratio of co-localization of EGFR-HA with LAMP1-positive lysosome in Vector (*n* = 12 fields) and RAB31^{Q65L} (*n* = 17 fields). **c** Up-panels, immunofluorescence of EGFR-HA (red), GFP-LC3 (green) and CD63 (magenta) in the indicated stable HeLa cells transiently expressing EGFR-HA and GFP-LC3 under SS. Low-panel, the ratio of co-localization of EGFR-HA with GFP-LC3-positive autophagosome in Vector (*n* = 7 fields) and RAB31^{Q65L} (*n* = 13 fields). **d** Immunofluorescence of EGFR-HA (green) with CD63 (red) in the indicated cancer cell lines stably expressing Flag-RAB31^{Q65L} and transiently expressing EGFR-HA under SS. **e** Somatic mutations of RAB31 in human cancers. The data were collected from COSMIC（the Catalogue Of Somatic Mutations In Cancer）and cBioPortal for Cancer Genomics. Mutations highlighted in red are the mutations similar to the constitutively active form Q65L. **f** Immunofluorescence of EGFR-HA (green) and CD63 (red) in the various Flag-RAB31 stable HeLa cells transiently expressing EGFR-HA under SS. **g** Immunofluorescence of EGFR-HA (red) and Flag-RAB31^{Q65L} (magenta) with CD63-GFP (green) in Flag-RAB31^{Q65L} stable HeLa cells transiently expressing EGFR-HA and CD63-GFP under SS using super-resolution structured illumination microscopy (SIM). All data are means ± S.D. Unpaired *t*-test was used to analyze the difference between the two groups. NS, no statistical significance. Scale bars, 10 μm.
