# Supplementary material for: RAB31 marks and controls an ESCRT-independent exosome pathway
Source: Cell Res. 2020 Sep 21;31(2):157–77. doi: 10.1038/s41422-020-00409-1 (PMC8027411; doi:10.1038/s41422-020-00409-1)
Supplement: Supplementary file 3 — Supplementary information, Fig. S3 [file 41422_2020_409_MOESM3_ESM.pdf]

Supplementary information, Fig. S3

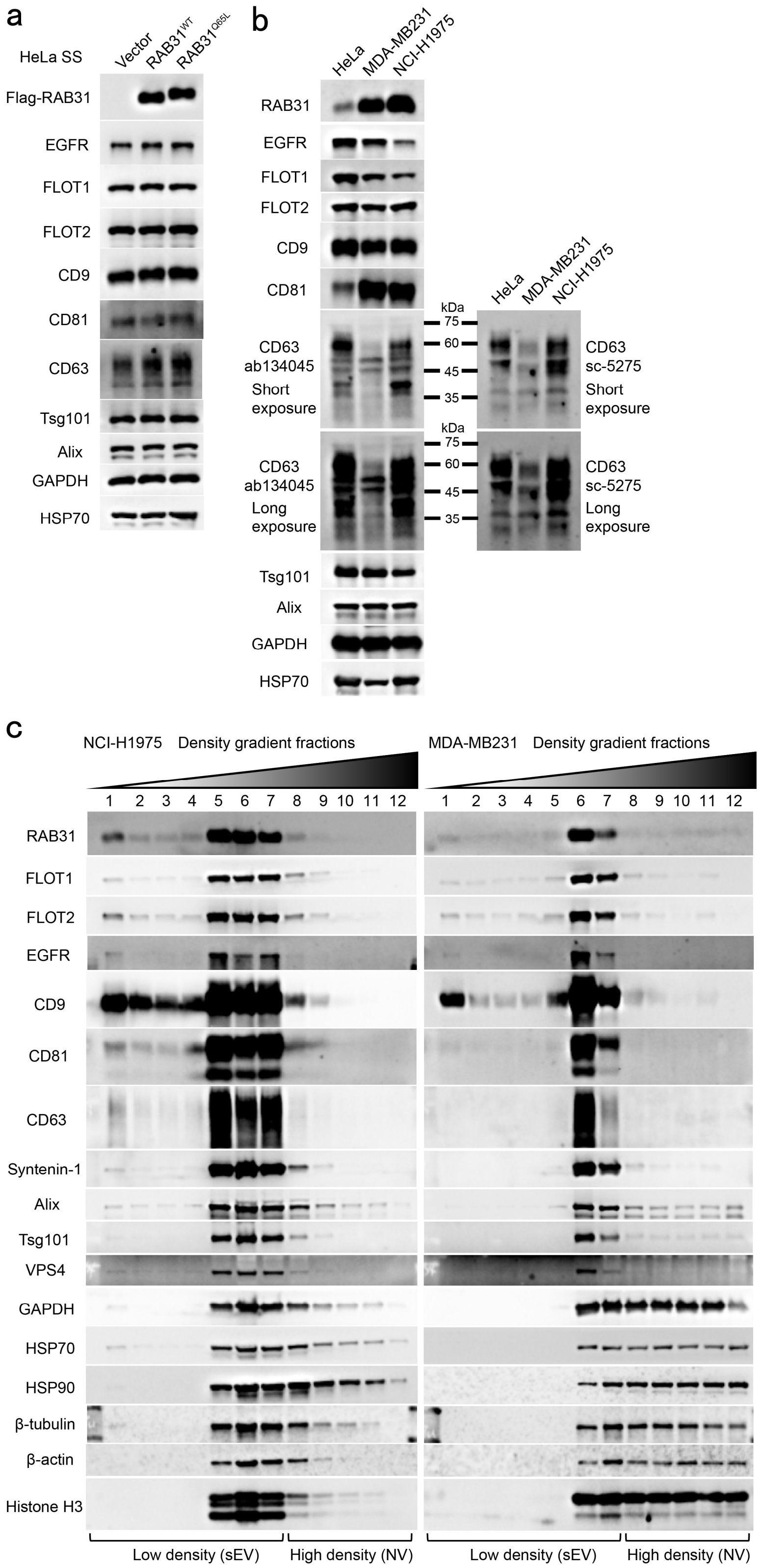

Supplementary information, Fig. S3. Expression pattern of three cancer cell lines. **a** Western blotting analyses of whole-cell lysates (WCL) from the indicated stable HeLa cells under serum starvation (SS). **b** Western blotting analyses of WCL from HeLa, MDA-MB231 and NCI-H1975 cells. Note: two CD63 antibodies (ab134045 and sc-5275) were used to detect the CD63 proteins with different glycosylation under both short and long exposure, as indicated. **c** Density gradient fractionation of small EVs isolated from the concentrated conditional media derived from NCI-H1975 and MDA-MB231 cells under SS. After flotation of sample in high-resolution iodixanol gradients, equal volumes of each fraction were loaded on SDS-PAGE gels, and membranes were blotted with the indicated antibodies. NV, non-vesicular; sEV, small EV.
