# Supplementary material for: RAB31 marks and controls an ESCRT-independent exosome pathway
Source: Cell Res. 2020 Sep 21;31(2):157–77. doi: 10.1038/s41422-020-00409-1 (PMC8027411; doi:10.1038/s41422-020-00409-1)
Supplement: Supplementary file 4 — Supplementary information, Fig. S4 [file 41422_2020_409_MOESM4_ESM.pdf]

Supplementary information, Fig. S4

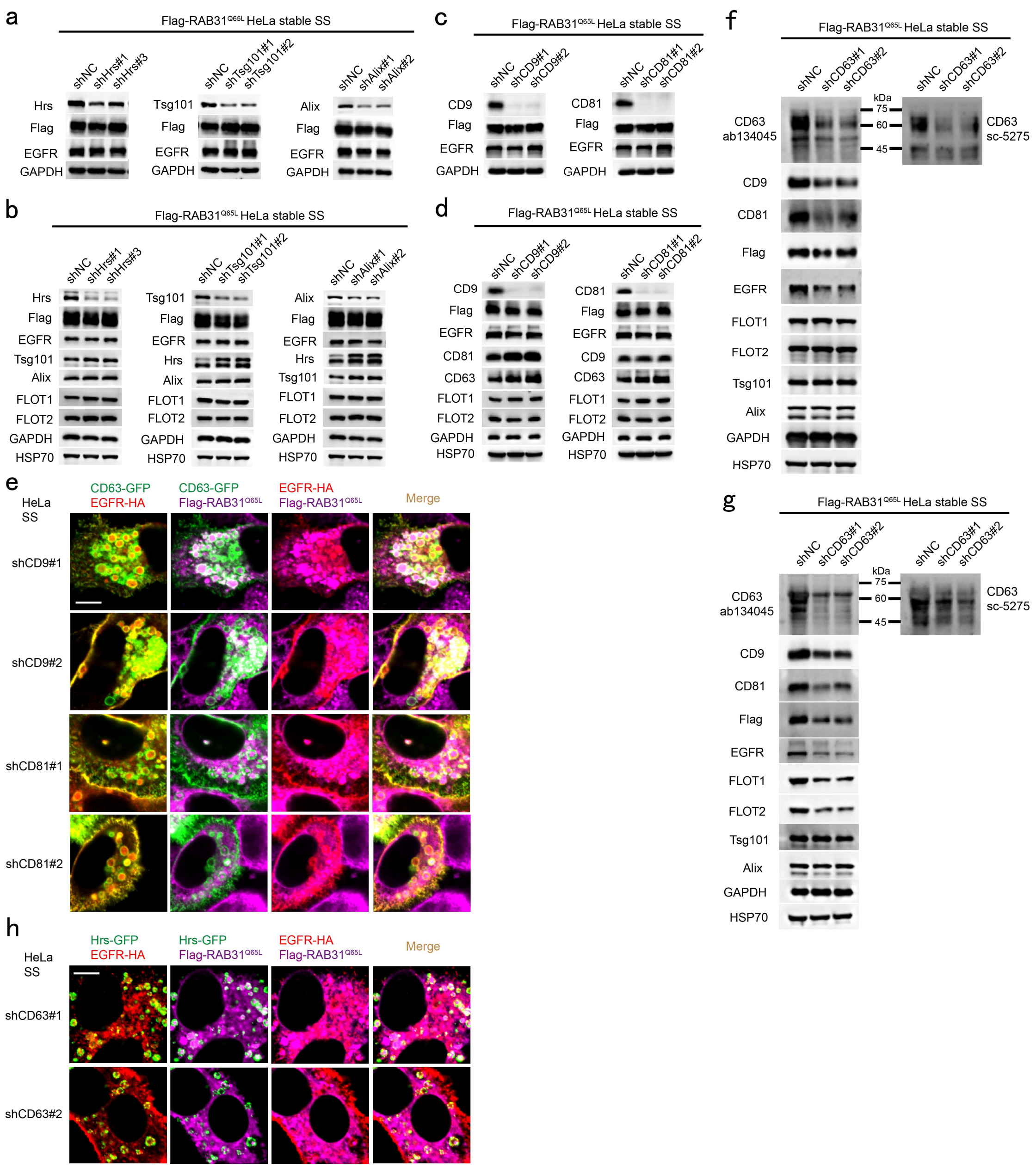

Supplementary information, Fig. S4. ESCRT, CD9 and CD81 are not required for the production of EGFR exosomes driven by RAB31^{Q65L}. a Western blotting analyses of whole-cell lysates (WCL) from Flag-RAB31^{Q65L} stable HeLa cells with the knockdown of Hrs, Tsg101 or Alix using two distinct shRNAs under serum starvation (SS). **b** Western blotting analyses of the concentrated conditional media from the cells used in **a** under SS. **c** Western blotting analyses of WCL from Flag-RAB31^{Q65L} stable HeLa cells with the knockdown of CD9 or CD81 using two distinct shRNAs under SS. **d** Western blotting analyses of the concentrated conditional media from the cells used in **c** under SS. **e** Immunofluorescence of EGFR-HA (red) and Flag-RAB31^{Q65L} (magenta) with CD63-GFP (green) in Flag-RAB31^{Q65L} stable HeLa cells stably expressing shCD9#1, shCD9#2, shCD81#1 or shCD81#2 and transiently expressing EGFR-HA and CD63-GFP under SS. **f** Western blotting analyses of WCL from Flag-RAB31^{Q65L} stable HeLa cells with the knockdown of CD63 using two distinct shRNAs under SS. **g** Western blotting analyses of the concentrated conditional media from the cells used in **f**. **h** Immunofluorescence of EGFR-HA (red) and Flag-RAB31^{Q65L} (magenta) with Hrs-GFP (green) in Flag-RAB31^{Q65L} stable HeLa cells stably expressing shCD63#1 or shCD63#2 and transiently expressing EGFR-HA and Hrs-GFP under SS. Scale bars, 10 μm.
