# Supplementary material for: RAB31 marks and controls an ESCRT-independent exosome pathway
Source: Cell Res. 2020 Sep 21;31(2):157–77. doi: 10.1038/s41422-020-00409-1 (PMC8027411; doi:10.1038/s41422-020-00409-1)
Supplement: Supplementary file 5 — Supplementary information, Fig. S5 [file 41422_2020_409_MOESM5_ESM.pdf]

Supplementary information, Fig. S5

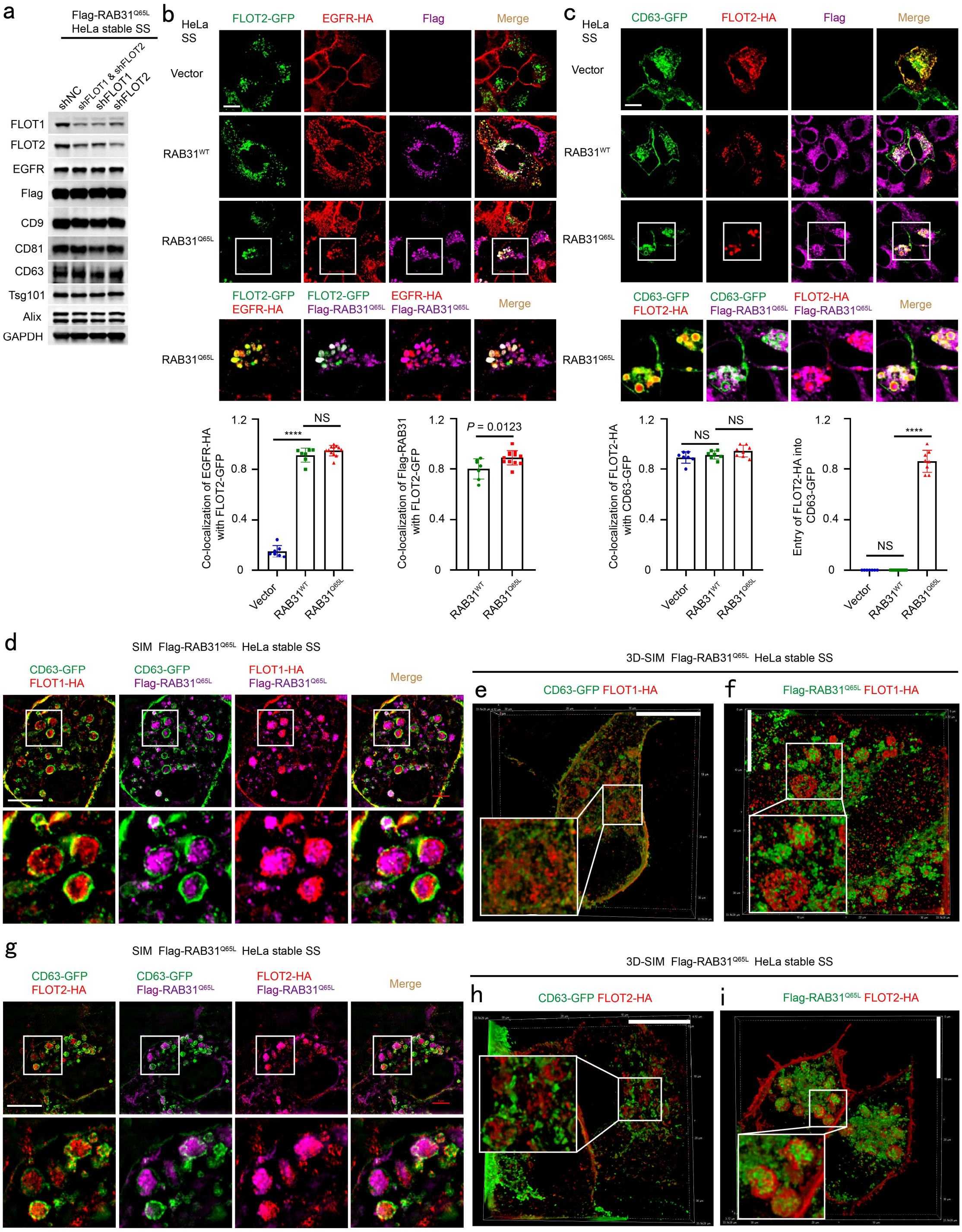

Supplementary information, Fig. S5. Active RAB31 engages FLOTs to drive EGFR-containing ILV formation depending on cholesterol and ceramide in lipid raft microdomains. a Western blotting analyses of whole-cell lysates from Flag-RAB31^{Q65L} stable HeLa cells with the knockdown of FLOT1, FLOT2, or both using shRNAs under serum starvation (SS). **b** Up-panels, immunofluorescence of EGFR-HA (red) and Flag-RAB31 (magenta) with FLOT2-GFP (green) in the indicated stable HeLa cells transiently expressing EGFR-HA and FLOT2-GFP under SS. Low-panel left, the ratio of co-localization of EGFR-HA with FLOT2-GFP-positive vesicle in Vector (n = 7 fields), RAB31^{WT} (n = 7 fields) and RAB31^{Q65L} (n = 12 fields). Low-panel right, the ratio of co-localization of Flag-RAB31 with FLOT2-GFP-positive vesicle in RAB31^{WT} (n = 7 fields) and RAB31^{Q65L} (n = 12 fields). **c** Up-panels, immunofluorescence of FLOT2-HA (red) and Flag-RAB31 (magenta) with CD63-GFP (green) in the indicated stable HeLa cells transiently expressing FLOT2-HA and CD63-GFP under SS. Low-panel left, the ratio of co-localization of FLOT2-HA with CD63-GFP-positive LE/MVE in Vector (n = 7 fields), RAB31^{WT} (n = 7 fields) and RAB31^{Q65L} (n = 8 fields). Low-panel right, the ratio of entry of FLOT2-HA into CD63-GFP-positive LE/MVE in Vector (n = 7 fields), RAB31^{WT} (n = 7 fields) and RAB31^{Q65L} (n = 8 fields). **d**, Immunofluorescence of FLOT1-HA (red) and Flag-RAB31^{Q65L} (magenta) with CD63-GFP (green) in Flag-RAB31^{Q65L} stable HeLa cells transiently expressing FLOT1-HA and CD63-GFP under SS using SIM. **e**, **f** Immunofluorescence of FLOT1-HA (red) with CD63-GFP (green) (**e**) or FLOT1-HA (red) with Flag-RAB31^{Q65L} (green) (**f**) in Flag-RAB31^{Q65L} stable HeLa cells transiently expressing FLOT1-HA and CD63-GFP under SS using 3D-SIM. **g**, Immunofluorescence of FLOT2-HA (red) and Flag-RAB31^{Q65L} (magenta) with CD63-GFP (green) in Flag-RAB31^{Q65L} stable HeLa cells transiently expressing FLOT2-HA and CD63-GFP under SS using SIM. **h**, **i** Immunofluorescence of FLOT2-HA (red) with CD63-GFP (green) (**h**) or FLOT2-HA (red) with Flag-RAB31^{Q65L} (green) (**i**) in Flag-RAB31^{Q65L} stable HeLa cells transiently expressing FLOT2-HA and CD63-GFP under SS using 3D-SIM. All data are means ± S.D. Unpaired *t*-test was used to analyze the difference between the two groups.

****$P < 0.0001$, NS, no statistical significance. Scale bars, 10 μm.
