# Supplementary material for: RAB31 marks and controls an ESCRT-independent exosome pathway
Source: Cell Res. 2020 Sep 21;31(2):157–77. doi: 10.1038/s41422-020-00409-1 (PMC8027411; doi:10.1038/s41422-020-00409-1)
Supplement: Supplementary file 6 — Supplementary information, Fig. S6 [file 41422_2020_409_MOESM6_ESM.pdf]

Supplementary information, Fig. S6

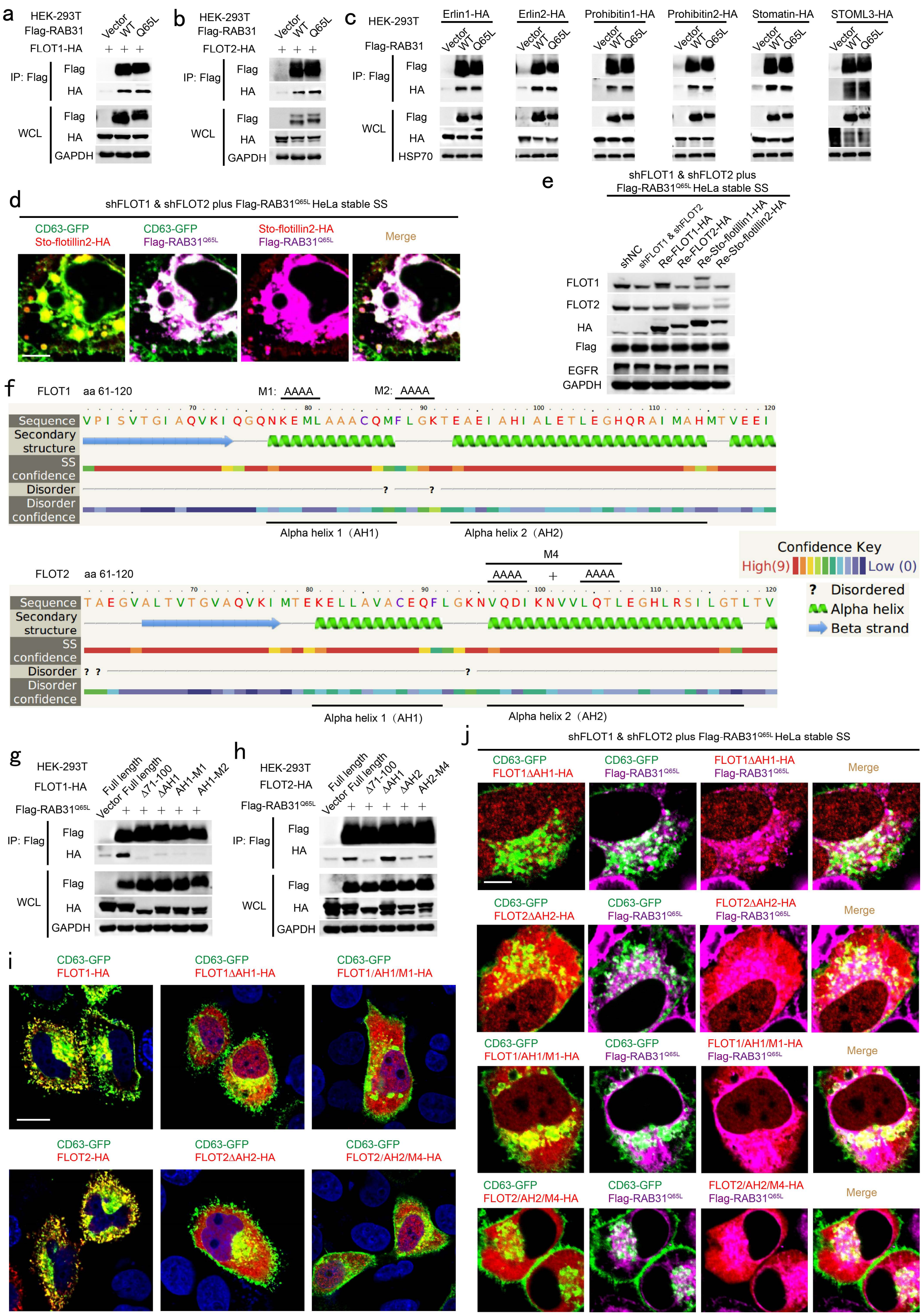

Supplementary information, Fig. S6. Active RAB31 interacts with the SPFH domain of FLOTs and drives MVE membrane budding to form ILVs via the Flotillin domain of FLOTs. a-c Western blotting analyses of whole-cell lysates (WCLs) and immunoprecipitates (IP) from HEK-293T cells co-expressing the indicated plasmids. **d** Immunofluorescence of Sto-flotillin2-HA (red) chimera and Flag-RAB31^{Q65L} (magenta) with CD63-GFP (green) in the indicated stable HeLa cells transiently expressing Sto-flotillin2-HA and CD63-GFP under serum starvation (SS). **e** Western blotting analyses of WCL from the indicated stable HeLa cells stably re-introduced with the indicated plasmids under SS. **f** The amino acid sequences and their mutants (M1, M2, M4) of AH1 or AH2, as well as secondary structures of amino acids 61-120 of FLOT1 or FLOT2. **g**, **h** Western blotting analyses of WCL and IP from HEK-293T cells co-expressing the indicated plasmids. **i** Immunofluorescence of FLOT1, FLOT2, FLOT1ΔAH1-HA, FLOT2ΔAH2-HA, FLOT1/AH1/M1-HA or FLOT2/AH2/M4-HA (red) with CD63-GFP (green) in normal HeLa cells transiently expressing the indicated plasmids. **j** Immunofluorescence of FLOT1ΔAH1-HA, FLOT2ΔAH2-HA, FLOT1/AH1/M1-HA or FLOT2/AH2/M4-HA (red) and Flag-RAB31^{Q65L} (magenta) with CD63-GFP (green) in the indicated stable HeLa cells transiently expressing the indicated plasmids under SS. Scale bars, 10 μm.
