# Supplementary material for: RAB31 marks and controls an ESCRT-independent exosome pathway
Source: Cell Res. 2020 Sep 21;31(2):157–77. doi: 10.1038/s41422-020-00409-1 (PMC8027411; doi:10.1038/s41422-020-00409-1)
Supplement: Supplementary file 7 — Supplementary information, Fig. S7 [file 41422_2020_409_MOESM7_ESM.pdf]

Supplementary information, Fig. S7

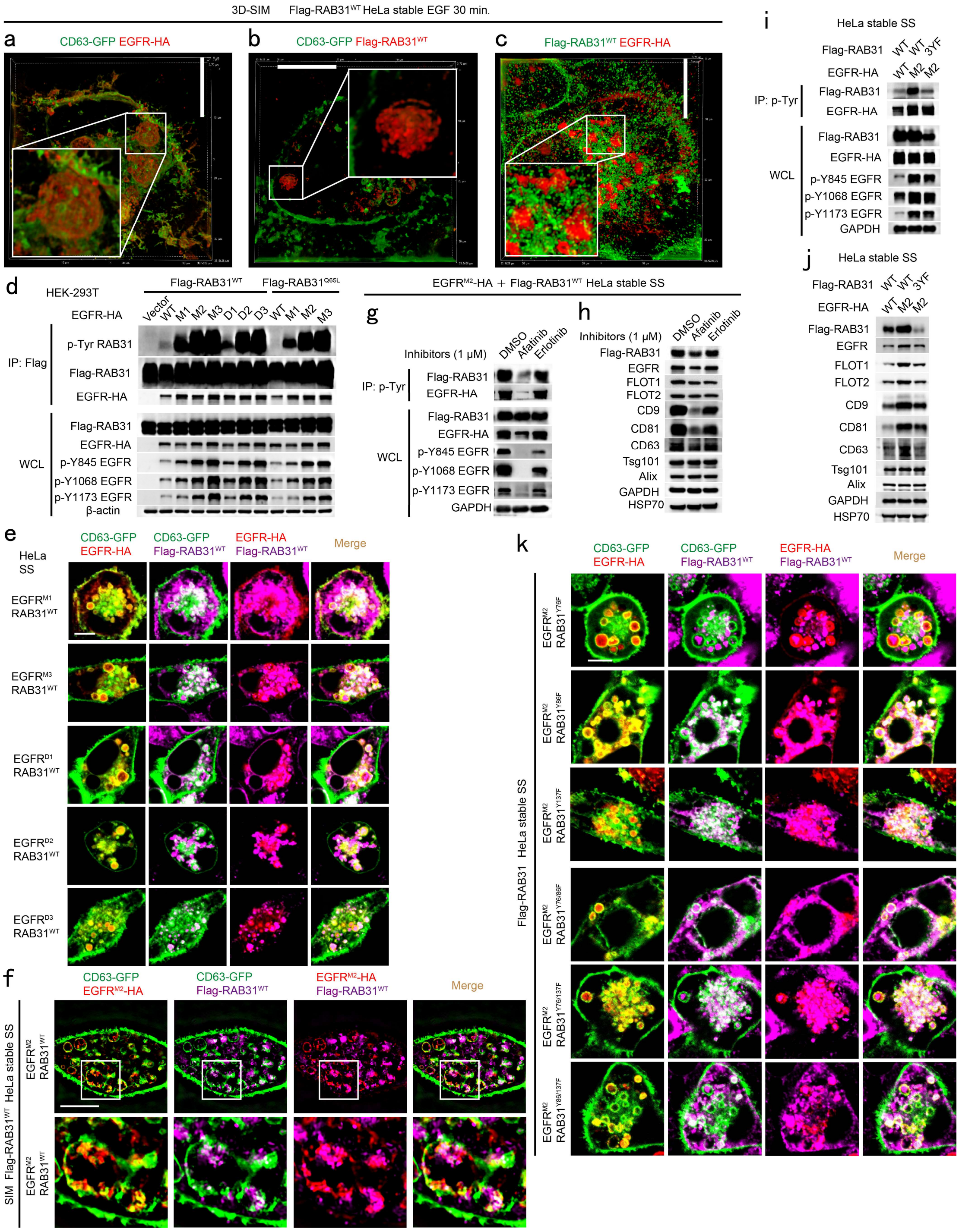

Supplementary information, Fig. S7. Tyrosine phosphorylation of RAB31 by active EGFR acts similar to its active form. a-c Immunofluorescence of EGFR-HA (red) with CD63-GFP (green) (**a**), Flag-RAB31^{WT} (red) with CD63-GFP (green) (**b**), and Flag-RAB31^{WT} (green) with EGFR-HA (red) (**c**) in Flag-RAB31^{WT} stable HeLa cells transiently expressing EGFR-HA and CD63-GFP treated with 100 ng/mL of EGF for 30 min using 3D-SIM. **d** Western blotting analyses of whole-cell lysates (WCL) and immunoprecipitates (IP) from HEK-293T cells co-expressing the indicated plasmids under serum starvation (SS). **e** Immunofluorescence of various EGFR-HA mutants (red) and Flag-RAB31^{WT} (magenta) with CD63-GFP (green) in Flag-RAB31^{WT} stable HeLa cells transiently expressing the EGFR-HA mutants and CD63-GFP under SS. **f** Immunofluorescence of EGFR^{M2}-HA (red) and Flag-RAB31^{WT} (magenta) with CD63-GFP (green) in Flag-RAB31^{WT} stable HeLa cells transiently expressing EGFR^{M2}-HA and CD63-GFP under SS using SIM. **g** Western blotting analyses of WCL and IP from the indicated stable HeLa cells treated with DMSO, afatinib or erlotinib under SS. **h** Western blotting analyses of the concentrated conditional media from the cells used in **g**. **i** Western blotting analyses of WCL and IP from the indicated stable HeLa cells under SS. **j** Western blotting analyses of the concentrated conditional media from the cells used in **i**. **k** Immunofluorescence of EGFR^{M2}-HA (red) and various mutants of Flag-RAB31 (magenta) with CD63-GFP (green) in Flag-RAB31 mutant stable HeLa cells transiently expressing EGFR^{M2}-HA and CD63-GFP under SS. Scale bars, 10 μm.
