# Supplementary material for: RAB31 marks and controls an ESCRT-independent exosome pathway
Source: Cell Res. 2020 Sep 21;31(2):157–77. doi: 10.1038/s41422-020-00409-1 (PMC8027411; doi:10.1038/s41422-020-00409-1)
Supplement: Supplementary file 8 — Supplementary information, Fig. S8 [file 41422_2020_409_MOESM8_ESM.pdf]

Supplementary information, Fig. S8

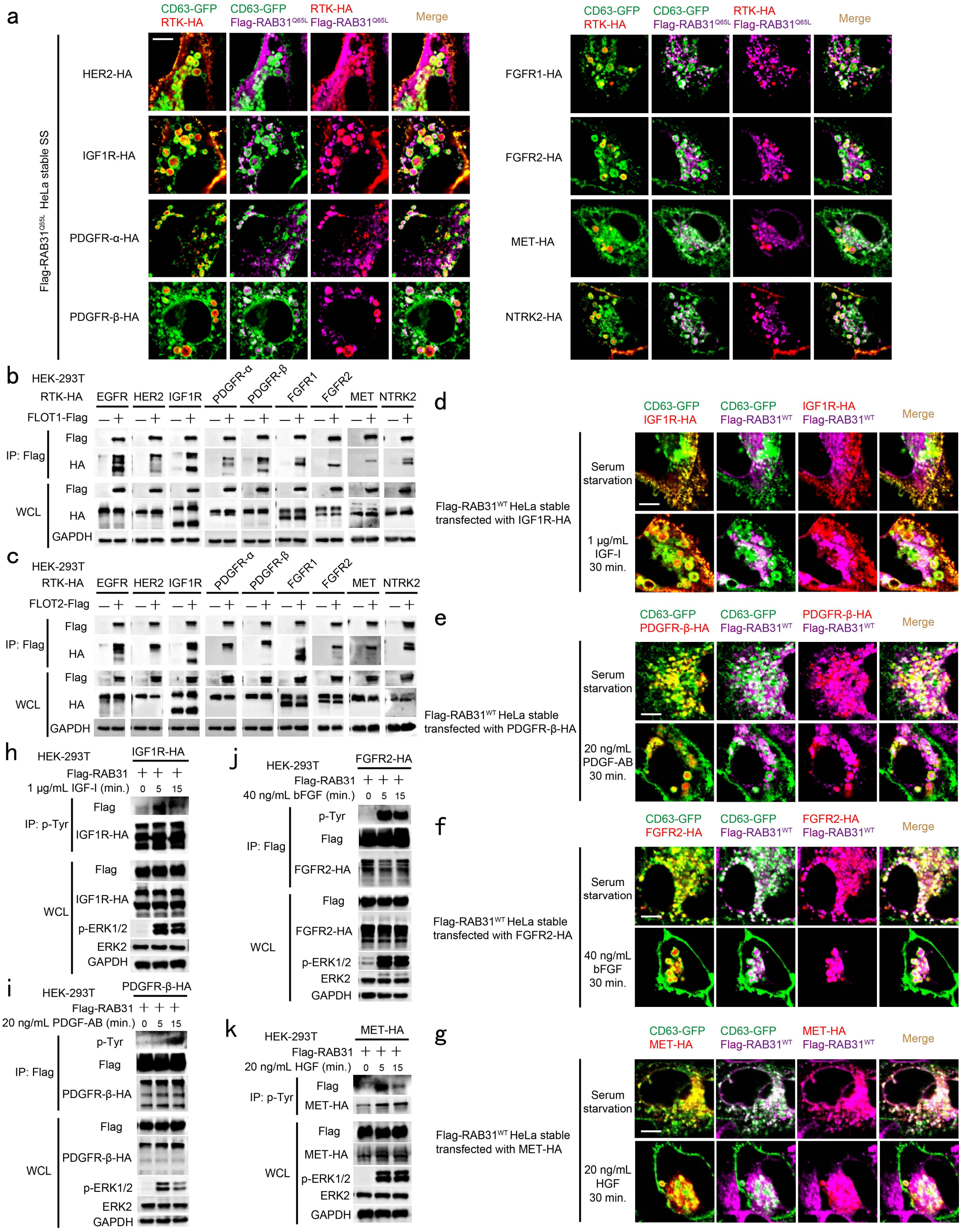

Supplementary information, Fig. S8. RTKs phosphorylate RAB31 to drive them entry CD63-positive MVE. a Immunofluorescence of the indicated RTKs-HA (red) and Flag-RAB31^{Q65L} (magenta) with CD63-GFP (green) in Flag-RAB31^{Q65L} stable HeLa cells transiently expressing RTK-HA and CD63-GFP under serum starvation (SS). **b**, **c** Western blotting analyses of whole-cell lysates (WCL) and immunoprecipitates (IP) from HEK-293T cells co-expressing the indicated plasmids. **d-g** Immunofluorescence of IGF1R-HA (**d**), PDGFR-β-HA (**e**), FGFR2-HA (**f**), or MET-HA (**g**) (red) and Flag-RAB31^{WT} (magenta) with CD63-GFP (green) in Flag-RAB31^{WT} stable HeLa cells transiently expressing RTK-HA and CD63-GFP under SS or with their corresponding ligands. **h-k** Western blotting analyses of WCL and IP from HEK-293T cells coexpressing Flag-RAB31^{WT} with IGF1R-HA (**h**), PDGFR-β-HA (**i**), FGFR2-HA (**j**), or MET-HA (**k**) under SS and treated with the indicated concentrations of their corresponding ligands. Scale bars, 10 μm.
