# Supplementary material for: RAB31 marks and controls an ESCRT-independent exosome pathway
Source: Cell Res. 2020 Sep 21;31(2):157–77. doi: 10.1038/s41422-020-00409-1 (PMC8027411; doi:10.1038/s41422-020-00409-1)
Supplement: Supplementary file 9 — Supplementary information, Fig. S9 [file 41422_2020_409_MOESM9_ESM.pdf]

Supplementary information, Fig. S9

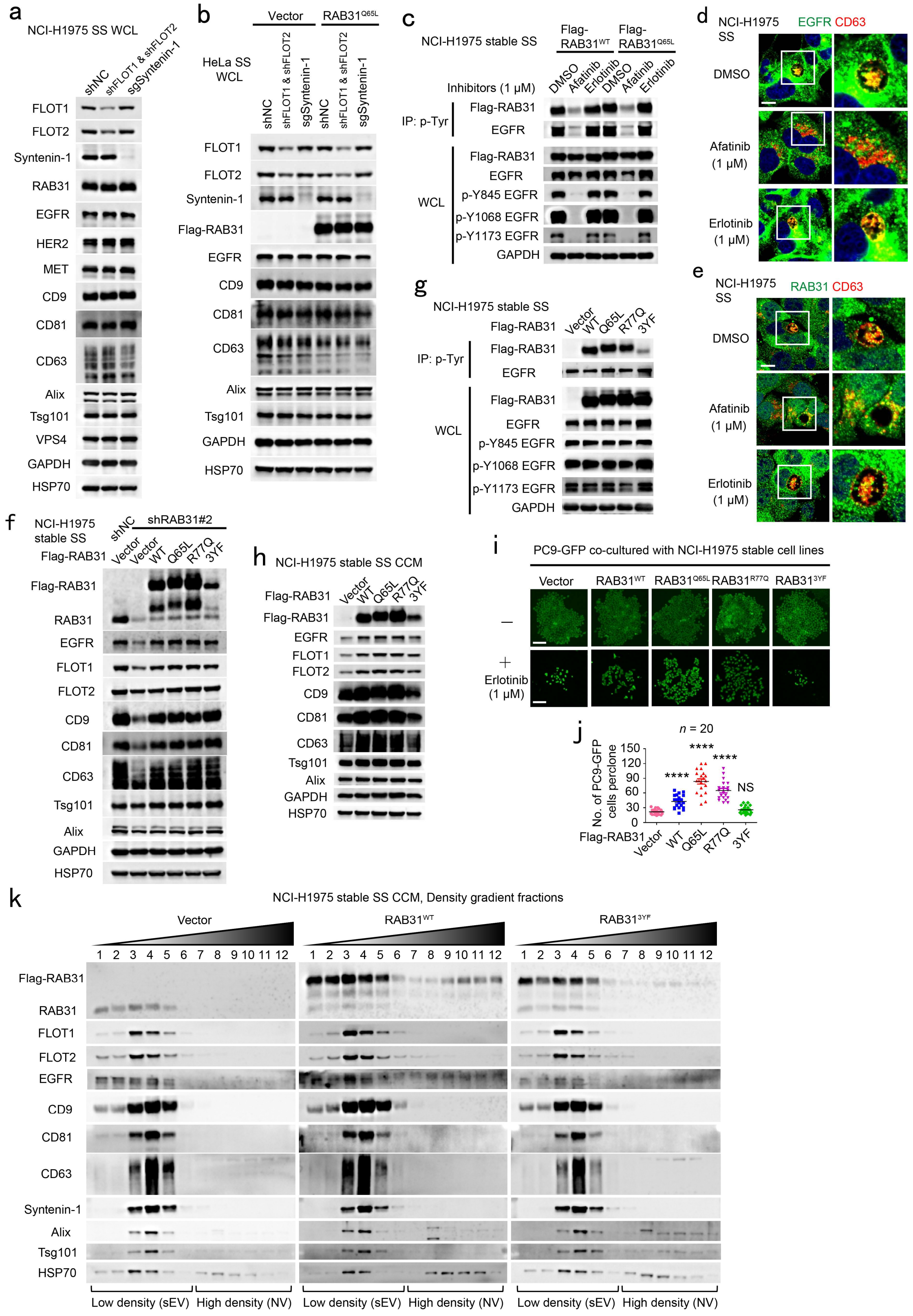

Supplementary information, Fig. S9. EGFR phosphorylates RAB31 to drive EGFR into exosomes and the exosomes promoted by RAB31 mediate resistance to erlotinib. a Western blotting analyses of whole-cell lysates (WCL) from the indicated stable NCI-H1975 cells under serum starvation (SS). **b** Western blotting analyses of WCL from the indicated stable HeLa cells under SS. **c** Western blotting analyses of WCL and immunoprecipitates (IP) from the indicated stable NCI-H1975 cells treated with DMSO, afatinib or erlotinib under SS. **d** Immunofluorescence of endogenous EGFR (green) and CD63 (red) in NCI-H1975 cells treated with afatinib or erlotinib under SS. **e** Immunofluorescence of endogenous RAB31 (green) and CD63 (red) in NCI-H1975 cells treated with afatinib or erlotinib under SS. **f** Western blotting analyses of WCL from the indicated stable NCI-H1975 cells under SS. **g** Western blotting analyses of WCL and IP from the indicated stable NCI-H1975 cells under SS. **h** Western blotting analyses of the concentrated conditional media from the indicated stable NCI-H1975 cells used in **g**. **i** Representative clone images of PC9-GFP cells co-cultured with the indicated stable NCI-H1975 cells without or with erlotinib. **j**, Quantification of the cell numbers of 20 colones in each group in **i**. Data are means ± S.D. of cell numbers of 20 clones in each group. Unpaired *t*-test was used to analyze the difference between the two groups. $^{****}P < 0.0001$, NS, no statistical significance. **k** Density gradient fractionation of small EVs isolated from the concentrated conditional media derived from stable NCI-H1975 cells under SS. After flotation of sample in high-resolution iodixanol gradients, equal volumes of each fraction were loaded on SDS-PAGE gels, and membranes were blotted with the indicated antibodies. NV, non-vesicular; sEV, small EV. Scale bars, 10 μm (**d** and **e**), 200 μm (**i**).
