# Supplementary material for: RAB31 marks and controls an ESCRT-independent exosome pathway
Source: Cell Res. 2020 Sep 21;31(2):157–77. doi: 10.1038/s41422-020-00409-1 (PMC8027411; doi:10.1038/s41422-020-00409-1)
Supplement: Supplementary file 10 — Supplementary information, Fig. S10 [file 41422_2020_409_MOESM10_ESM.pdf]

Supplementary information, Fig. S10

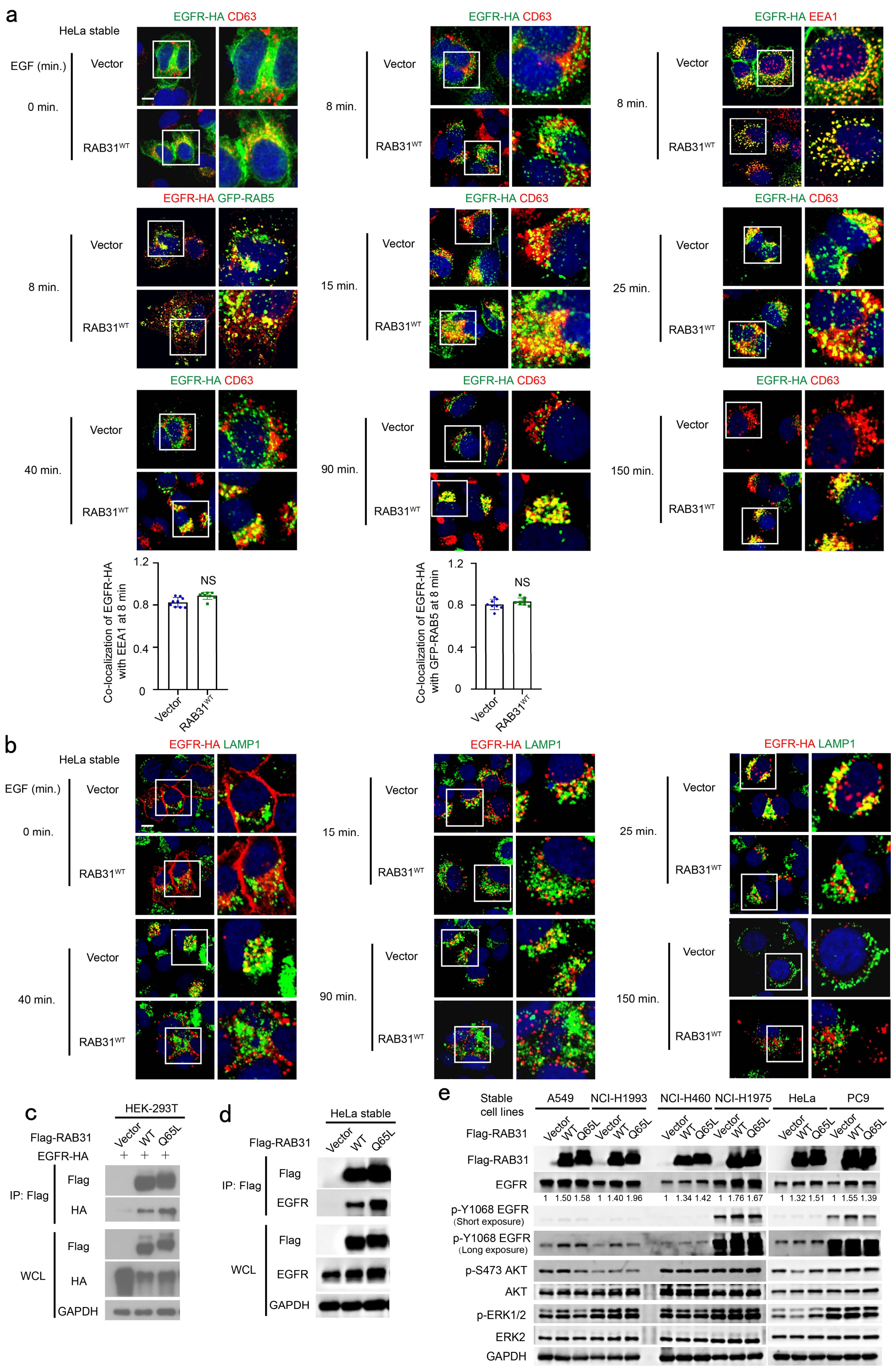

Supplementary information, Fig. S10. RAB31 sequesters EGFR in CD63-positive MVEs. **a** Up-panels Immunofluorescence of EGFR-HA (green) with CD63 (red), EGFR-HA (green) with EEA1 (red) or EGFR-HA (red) with GFP-RAB5 (green) in the indicated stable HeLa cells transiently expressing EGFR-HA treated with 100 ng/mL of EGF at the indicated times. Low-panel left, the ratio of co-localization of EGFR-HA with EEA1-positive vesicle in Vector (n = 9 fields) and $RAB31^{WT}$ (n = 8 fields) at 8 min of EGF treatment. Low-panel right, the ratio of co-localization of EGFR-HA with GFP-RAB5-positive vesicle in Vector (n = 8 fields) and $RAB31^{WT}$ (n = 8 fields) at 8 min of EGF treatment. Data are means ± S.D. Unpaired t-test was used to analyze the difference between the two groups. NS, no statistical significance. **b** Immunofluorescence of EGFR-HA (red) with LAMP1 (green) in the indicated stable HeLa cells transiently expressing EGFR-HA treated with 100 ng/mL of EGF at the indicated times. **c** Western blotting analyses of whole-cell lysates (WCL) and immunoprecipitates (IP) from HEK-293T cells co-expressing EGFR-HA and Flag-RAB31. **d** Western blotting analyses of WCL and IP from the indicated stable HeLa cells. **e** Western blotting analyses of WCL from various cancer cell lines stably expressing Flag-RAB31. Scale bars, 10 μm.
