# Supplementary material for: RAB31 marks and controls an ESCRT-independent exosome pathway
Source: Cell Res. 2020 Sep 21;31(2):157–77. doi: 10.1038/s41422-020-00409-1 (PMC8027411; doi:10.1038/s41422-020-00409-1)
Supplement: Supplementary file 11 — Supplementary information, Fig. S11 [file 41422_2020_409_MOESM11_ESM.pdf]

Supplementary information, Fig. S11

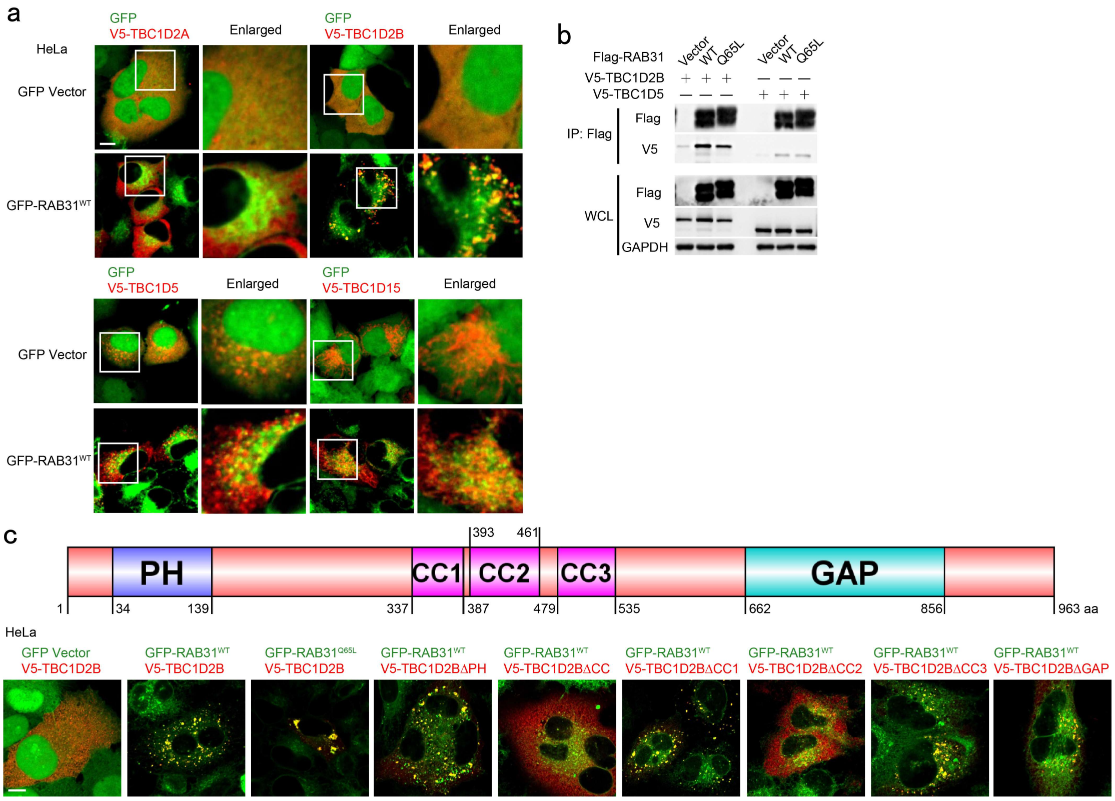

Supplementary information, Fig. S11. RAB31 recruits TBC1D2B to late endosomes and MVEs. a Immunofluorescence of V5-TBC1D2A, V5-TBC1D2B, V5-TBC1D5 or V5-TBC1D15 (red) with GFP-RAB31^{WT} (green) in the indicated stable HeLa cells transiently expressing the indicated plasmids. **b** Western blotting analyses of whole-cell lysates (WCL) and immunoprecipitates (IP) from HEK-293T cells co-expressing the indicated plasmids. **c** Up-panel, Schematic depicting the domains of TBC1D2B. Low-panels, immunofluorescence of V5-TBC1D2B or its truncations (red) with GFP-RAB31 (green) in GFP-RAB31 stable HeLa cells transiently expressing the indicated plasmids. PH, Pleckstrin homolgy domain, CC, coiled-coil domain, GAP, GTPase-activating protein domain. Scale bars, 10 μm.
